# Negative feedback between PTH1R and IGF1 through the Hedgehog pathway in mediating craniofacial bone remodeling

**DOI:** 10.1172/jci.insight.183684

**Published:** 2024-12-17

**Authors:** Yi Fan, Ping Lyu, Jiahe Wang, Yali Wei, Zucen Li, Shiwen Zhang, Takehito Ouchi, Junjun Jing, Quan Yuan, Clifford J. Rosen, Chenchen Zhou

**Affiliations:** 1 State Key Laboratory of Oral Diseases, National Center for Stomatology, National Clinical Research Center for Oral Diseases,; 2 Department of Cariology and Endodontics,; 3 Department of Pediatric Dentistry, and; 4 Department of Oral Implantology, West China Hospital of Stomatology, Sichuan University, Chengdu, China.; 5 Department of Physiology, Tokyo Dental College, Tokyo, Japan.; 6 Maine Medical Center Research Institute, Scarborough, Maine, USA.

**Keywords:** Bone biology, Development, Bone development, G protein&ndash;coupled receptors, Osteoclast/osteoblast biology

## Abstract

Regeneration of orofacial bone defects caused by inflammation-related diseases or trauma remains an unmet challenge. Parathyroid hormone 1 receptor (PTH1R) signaling is a key mediator of bone remodeling whereas the regulatory mechanisms of PTH1R signaling in oral bone under homeostatic or inflammatory conditions have not been demonstrated by direct genetic evidence. Here, we observed that deletion of PTH1R in Gli1^+^ progenitors led to increased osteogenesis and osteoclastogenesis. Single-cell and bulk RNA-Seq analysis revealed that PTH1R suppressed the osteogenic potential of Gli1^+^ progenitors during inflammation. Moreover, we identified upregulated IGF1 expression upon PTH1R deletion. Dual deletion of IGF1 and PTH1R ameliorated the bone-remodeling phenotypes in PTH1R-deficient mice. Furthermore, in vivo evidence revealed an inverse relationship between PTH1R and Hedgehog signaling, which was responsible for the upregulated IGF1 production. Our work underscored the negative feedback between PTH1R and IGF1 in craniofacial bone turnover and revealed mechanisms modulating orofacial bone remodeling.

## Abstract 2

PTH1R couples bone formation and bone resorption by negatively regulating Hedgehog signaling and IGF1 production.

## Graphical abstract

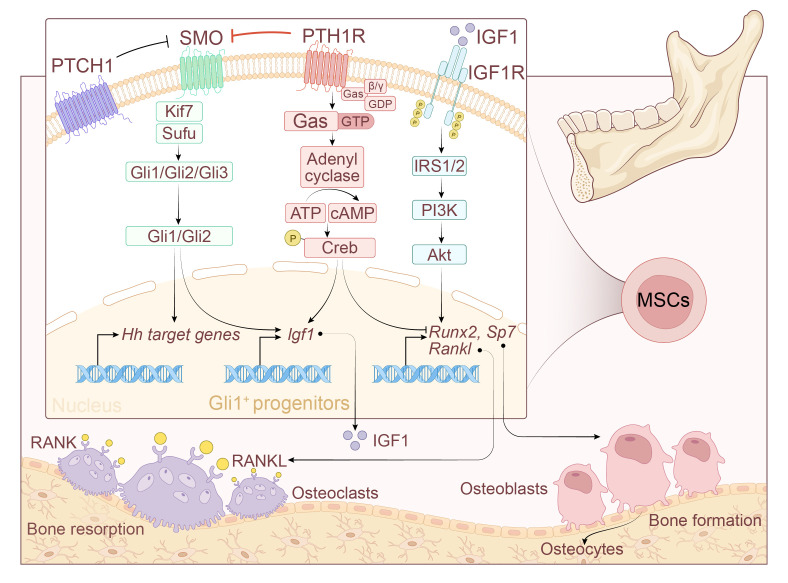

## Introduction

Oral inflammatory diseases such as periodontitis and periapical diseases affect more than 1 billion people globally (1). They are the most common cause of tooth loss in adults. Globally, oral inflammatory diseases cause high financial and health burdens and have an undeniable negative impact on quality of life (2). Inflammatory conditions progressively destroy the periodontal tissues, resulting in the loss of periodontal ligament (PDL) attachment and resorption of surrounding bone (3). Regeneration of craniofacial bone remains an unmet challenge in clinical practice (4). Mesenchymal stem cell–based (MSC-based) regeneration strategies present great potential for healing bone and dental defects (5). Orofacial MSCs play an essential role in the development, maintenance, and repair of the bony tissue to its original architecture and function (6, 7). Stem cells expressing Acta2, Axin2, Gli1, Prrx1, PTHrP, Sp7, Lepr, and Plap1 have been found in the craniofacial region (8–14). In addition, Gli1^+^ cells have recently been identified as important MSCs that reside in mouse periodontium and give rise to bone, PDL, and cementum. However, their distinct regulatory mechanism remains elusive (11).

Parathyroid hormone 1 receptor (PTH1R) signaling is a major regulator of skeletal development, bone remodeling, and mineral ion homeostasis through multiple actions on bone and kidney (15). It is activated by parathyroid hormone (PTH) and parathyroid hormone–related peptide (PTHrP) (16). Human PTH1R mutations not only affect endochondral bones but also are associated with multiple disorders in the craniofacial regions, such as skeletal malformations, ankylosis, and distorted teeth (17, 18). Analysis of transgenic mouse models suggests that PTH1R signaling functions in the maintenance of tooth root formation, tooth eruption, and alveolar bone formation and regeneration (8, 9, 12, 19). Yet, the phenotypical findings in PTH1R-deficient mice are varied owing to the diverse function of PTH1R in different mesenchymal progenitors. For instance, global ablation of PTH1R and deletion of PTH1R in Sp7- or PTHrP-expressing mesenchymal progenitors caused accelerated mandibular bone and cementum mineralization (9, 19, 20). However, mice generated by gene targeting to delete PTH1R in Prrx1^+^ and Lepr^+^ progenitors showed decreased alveolar bone mineral density because of lower bone formation rate (8, 12). Thus, the specific regulatory mechanism of PTH1R in orofacial mesenchymal progenitors remains unexplained. In this study, we evaluated the cell fate of Gli1^+^ mesenchymal progenitors during craniofacial development. We conditionally ablated PTH1R to dissect its role in oral bone remodeling and PDL turnover. Our findings revealed that PTH1R deficiency accelerated osteogenic and osteoclastogenic activities, which led to decreased alveolar bone volume and PDL malformation. Furthermore, we assessed the regulatory mechanisms of PTH1R signaling in inflammation-related bone disease and found negative feedback between PTH1R and IGF1 in determining the cell fate of Gli1^+^ mesenchymal progenitor cells during oral bone and periodontium development and repair. We have also identified the pivotal role of Hedgehog signaling in mediating elevated IGF1 levels due to PTH1R deletion in oral bone. These findings point to a signaling pathway that could enable innovations in therapeutic strategies for regenerating orofacial tissues resulting from inflammatory bone diseases, developmental defects, and trauma.

## Results

### Gli1^+^ MSCs participate in craniofacial tissue development.

We first performed a lineage tracing experiment using *Gli1^CreER^ Rosa26^Ai14^* mice to map the cell fate of Gli1^+^ MSCs at different developmental stages. Analysis of mice at postnatal day 14 (P14) after a tamoxifen pulse at P7 revealed that Gli1^+^ cells were located in the dental mesenchyme and gave rise to the entire radicular pulp, periostin^+^ PDL cells, and Runt-related transcription factor 2–positive (RUNX2^+^) osteoblasts in the oral bone (Supplemental Figure 1, A–C; supplemental material available online with this article; https://doi.org/10.1172/jci.insight.183684DS1). The descendants of Gli1^+^ MSCs marked at P14 (Gli1-P14 cells) were located within 2/3 of the apical portion of the radicular pulp, PDL, osteoblasts, and osteocytes in the surrounding bone at 6 weeks old (Supplemental Figure 1, D–F). After 21 days of chase at P21, Gli1-P21 cells were present in all PDL cells and osteoblasts, osteocytes (23.59% ± 1.95%) in bone, and the apical third of the radicular pulp (Supplemental Figure 1, G–I). After 42 days of chase at P63, Gli1-P21 cells continued to contribute substantially to PDL cells, cementoblasts (77.57% ± 1.18%), and osteocytes (34.61% ± 0.67%) in cryptal bone. The proportion of *Rosa26^Ai14^*-positive cells expanded in the radicular and coronal pulp (Supplemental Figure 1, J–L). We also analyzed the long-term clonal maintenance of Gli1^+^ lineage cells at P200. The results showed that these cells retained stemness in PDL and alveolar bone across the development stage and adulthood. The ratio of Gli1^+^ cells in osteocytes increased to 44.89% ± 3.75% (Supplemental Figure 1, M–O). Therefore, Gli1^+^ MSCs were the mesenchymal progenitors for PDL cells, cementoblasts, radicular pulp, and alveolar cryptal osteoblasts and -cytes in vivo.

### PTH1R deletion causes decreased oral bone volume and PDL malformation.

**Figure 1:**
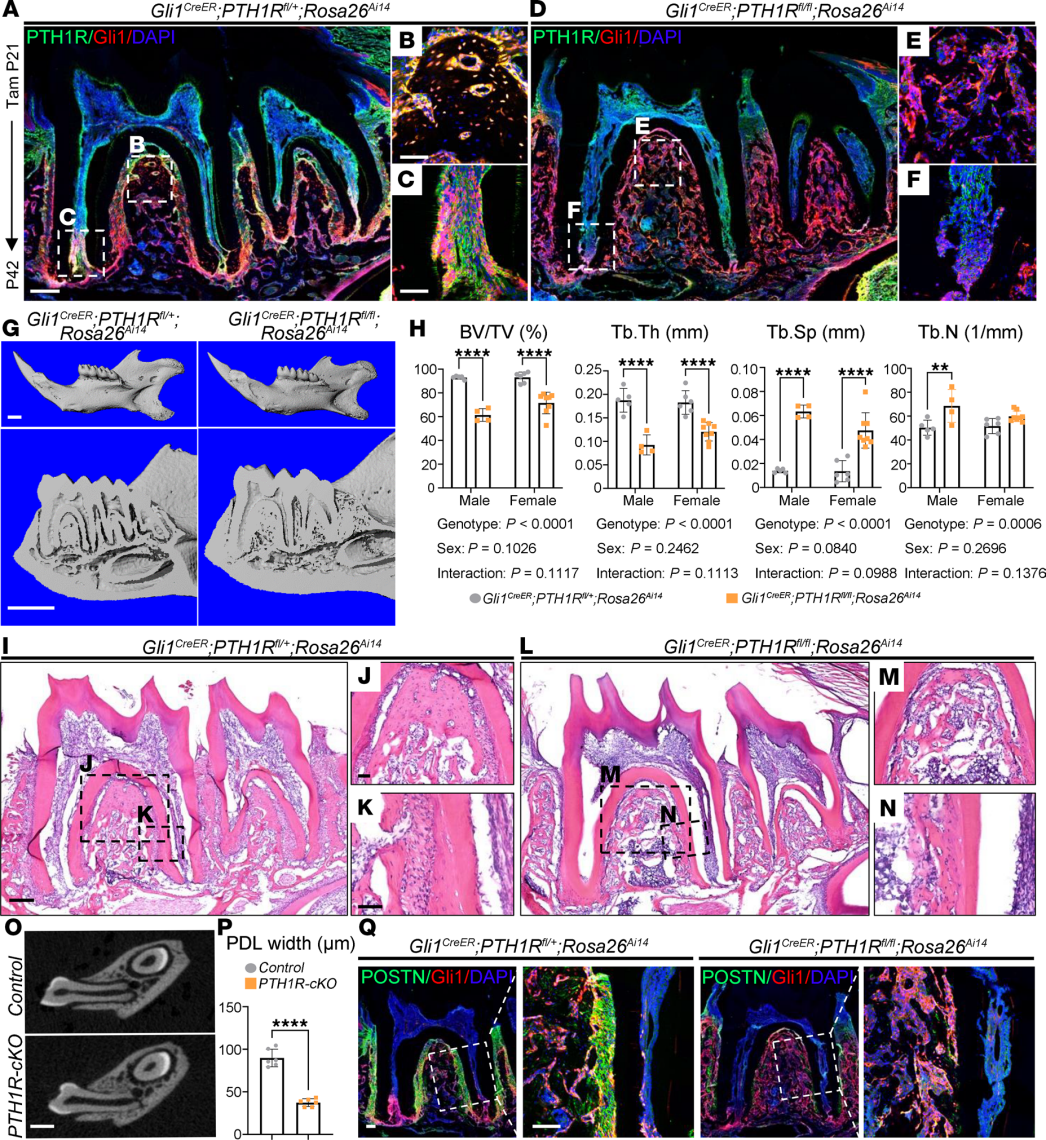
PTH1R deletion causes decreased alveolar bone volume and PDL malformation. (**A**–**F**) Immunofluorescence staining of PTH1R of *Gli1^CreER^ PTH1R^fl/+^ Rosa26^Ai14^* and *Gli1^CreER^ PTH1R^fl/fl^ Rosa26^Ai14^* female mice at P42. (**B**, **C**, **E**, and **F**) Higher magnification of boxed regions, respectively. *n* = 3. (**G**) Three-dimensional micro-CT reconstruction of *Gli1^CreER^ PTH1R^fl/+^ Rosa26^Ai14^* and *Gli1^CreER^ PTH1R^fl/fl^ Rosa26^Ai14^* mandibles at P42. *n* = 5 for male and *n* = 6 for female *Gli1^CreER^ PTH1R^fl/+^ Rosa26^Ai14^* mice. *n* = 4 for male and *n* = 8 for female *Gli1^CreER^ PTH1R^fl/fl^ Rosa26^Ai14^* mice. (**H**) Quantitative micro-CT analysis of BV/TV, Tb.Th, Tb.Sp, and Tb.N of both sexes in each genotype. (**I** and **L**) HE staining of *Gli1^CreER^ PTH1R^fl/+^ Rosa26^Ai14^* and *Gli1^CreER^ PTH1R^fl/fl^ Rosa26^Ai14^* female mice at P42. (**J** and **M**) Enlarged boxed areas of alveolar root furcation of mandibular first molar showed substantially reduced bone volume in PTH1R-cKO mice. (**K** and **N**) Higher magnification of PDL region showed the PDL space was replaced by bony tissue in PTH1R-cKO mice. *n* = 3. (**O**) Two-dimensional micro-CT images of coronal sections showed narrowed PDL space in PTH1R-cKO mice. *n* = 6. (**P**) Quantitative analysis of PDL width. *n* = 6. Female mice were used. (**Q**) Immunofluorescence staining of periostin (POSTN) of *Gli1^CreER^ PTH1R^fl/+^ Rosa26^Ai14^* and *Gli1^CreER^ PTH1R^fl/fl^ Rosa26^Ai14^* female mice at P42. Boxed areas are shown at higher magnification. *n* = 3. Scale bar = 200 μm (**A**, **B**, **I**, and **L**), 50 μm (**B**, **C**, **J**, and **K**), 500 μm (**G** and **O**), and 100 μm (**Q**). Significance is determined using unpaired 2-sided Student’s *t* tests in **H** or using 2-way ANOVA with Tukey’s correction for multiple comparisons in **D**. Data are mean ± SEM. **P < 0.01, ****P < 0.0001.

PTH1R is a key regulator in bone development and turnover wherein it directs the fate of MSCs (21). PTH1R immunoreactivity was broadly detected in the mesenchyme region of dental pulp, PDL, and alveolar bone and was highly colocalized with Gli1^+^ lineage cells (Supplemental Figure 2A). We deleted PTH1R in Gli1^+^ MSCs to specifically define the role of PTH1R in periodontium tissue. *Gli1^CreER^ PTH1R^fl/fl^ Rosa26^Ai14^* (PTH1R-cKO) mice were treated with tamoxifen at P21 and analyzed at P42. Immunostaining verified the efficiency of PTH1R deletion in targeted cells (Figure 1, A–F).

Three-dimensional reconstructive images from micro-CT revealed a porous microarchitecture of alveolar trabecular bone in PTH1R-cKO mice. Significantly decreased bone volume/tissue volume (BV/TV) and trabecular thickness (Tb.Th) were observed along with higher trabecular separation (Tb.Sp) and trabecular number (Tb.N) in the furcation area of the mandibular first molars in both male and female PTH1R-cKO mice (Figure 1, G and H). Hematoxylin and eosin (HE) staining showed that PTH1R-cKO mandibles had a slender trabecular bone structure with enlarged trabecular space compared with the well-organized bony structure observed in control littermates (Figure 1, I–N). Of note, quantitative micro-CT analyses revealed that PTH1R-cKO mice failed to form PDL tissue, as indicated by reduced PDL width and loss of PDL structure (Figure 1, O and P). Moreover, we performed immunostaining for periostin, a marker for PDL fibroblasts (22). Gli1^+^ PDL cells abundantly expressed periostin in control littermates, whereas only a low number of cells expressed periostin in PTH1R-cKO mice, implying the impaired PDL differentiation of Gli1^+^ progenitor cells in the absence of PTH1R (Figure 1Q).

### Deletion of PTH1R in Gli1^+^ lineage cells stimulates bone remodeling.

**Figure 2:**
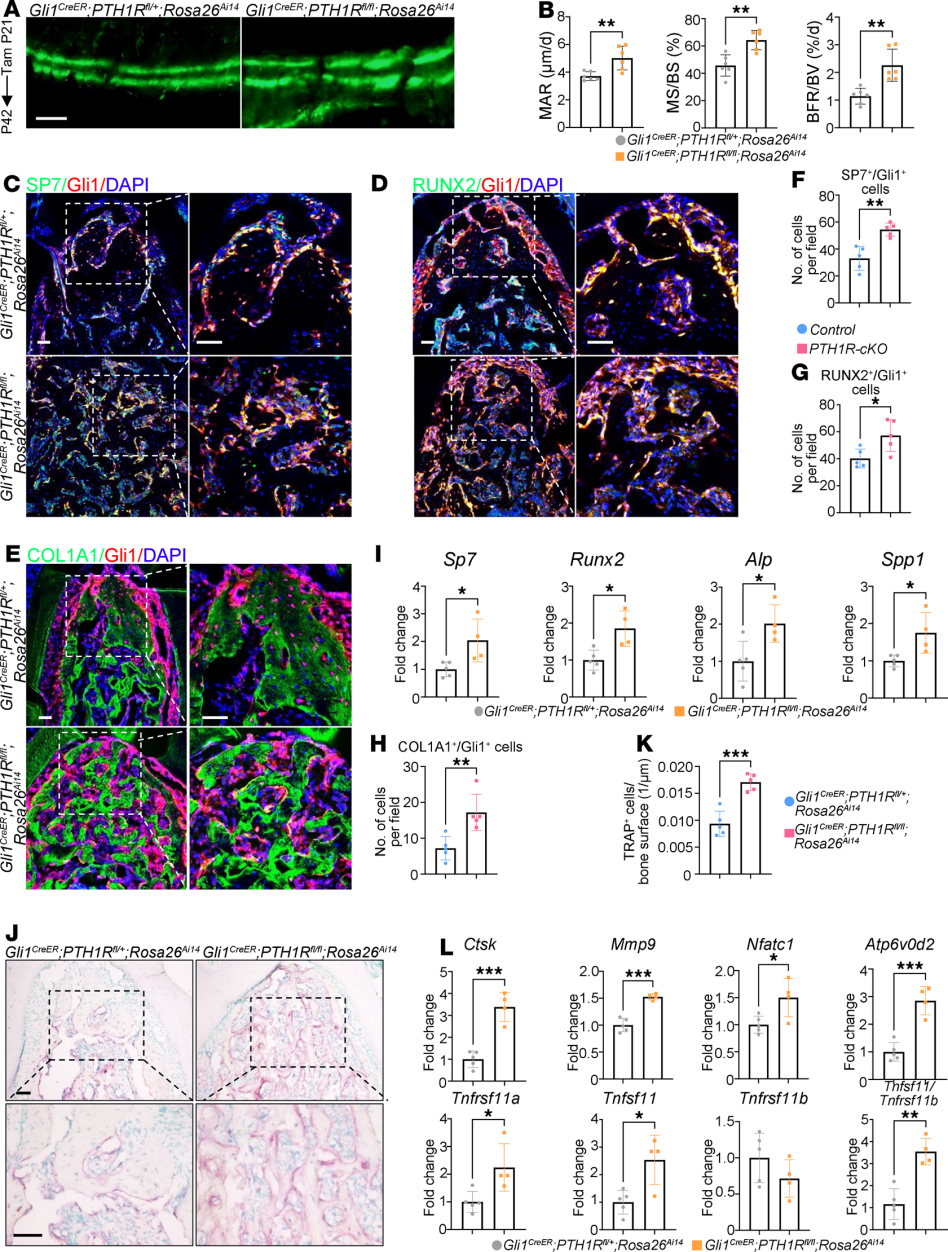
Deletion of PTH1R in Gli1^+^ lineage cells stimulates bone remodeling. (**A**) Double calcein labeling in the alveolar bone region of control and PTH1R-cKO mice at P42. *n* = 6. (**B**) Histomorphometry analysis of dynamic bone formation parameters at P42. *n* = 6. (**C**–**H**) Immunofluorescence staining of SP7 (**C**), RUNX2 (**D**), and COL1A1 (**E**) and quantification of SP7^+^Gli1^+^ (**F**), RUNX2^+^Gli1^+^ (**G**), and COL1A1^+^Gli1^+^ (**H**) cell number in PTH1R-cKO mice at P42. Boxed areas are shown at higher magnification. *n* = 5. (**I**) RT-qPCR analysis of osteogenesis-related gene expression (*Sp7, Runx2, Alp, Spp1*) in mandibles of PTH1R-cKO mice and control littermates at P42. *n* = 5 for control and *n* = 4 for PTH1R-cKO. (**J**) TRAP staining revealed increased TRAP^+^ osteoclast number in PTH1R-cKO mice at P42. Boxed areas are shown at higher magnification. *n* = 5. (**K**) Quantification of TRAP^+^ osteoclast number/bone surface. *n* = 5. (**L**) RT-qPCR analysis of osteoclastogenesis-related gene expression (*Ctsk, Mmp9, Nfatc1, Atp6v0d2, Tnfrsf11a, Tnfsf11, Tnfrsf11b, Tnfsf11/Tnfrsf11b*) in mandibles of PTH1R-cKO mice and control littermates at P42. *n* = 5 for control and *n* = 4 for PTH1R-cKO. Scale bar = 25 μm (**A**), 50 μm (**C**–**E** and **J**). Data were all obtained in female mice. Significance is determined using unpaired 2-sided Student’s *t* tests. Data are mean ± SEM. *P < 0.05, **P < 0.01, ***P < 0.001.

We further explored the mechanisms underlying decreased oral bone mass with dynamic histomorphometry analysis. This revealed significant increases in mineral apposition rate (MAR), mineral surface/bone surface (MS/BS), and bone formation rate/bone volume (BFR/BV) in PTH1R-cKO alveolar bone at P42 (Figure 2, A and B). We also analyzed the bone histomorphometry at P112. Increased MAR, MS/BS, and BFR/BV were observed in PTH1R-cKO mice when compared with age-matched controls (Supplemental Figure 3). Subsequently, we performed immunofluorescence staining to characterize the osteogenic activity of Gli1^+^ lineage cells. Immunoreactivity to SP7, RUNX2, and collagen type I α1 chain (COL1A1) were markedly elevated in Gli1^+^ cells upon PTH1R deletion (Figure 2, C–H). Moreover, real-time quantitative PCR (RT-qPCR) analysis of RNA isolated from alveolar bone showed that osteogenesis-related markers, including *Sp7*, *Runx2*, alkaline phosphatase (*Alp*), and secreted phosphoprotein 1 (*Spp1*), were markedly upregulated in PTH1R-cKO mice when compared with the controls (Figure 2I). We next cultured orofacial mesenchymal stem cells (OMSCs) from *PTH1R^fl/fl^ Rosa26^Ai14^* and *Gli1^CreER^ PTH1R^fl/fl^ Rosa26^Ai14^* mice. After osteogenic induction for 7 and 14 days, PTH1R-ablated OMSCs showed significantly increased ALP and alizarin red staining (ARS) intensity, respectively (Supplemental Figure 4, A and B). Gene expression analysis revealed upregulation of bone formation markers in PTH1R-deficient OMSCs, such as *Sp7*, *Runx2*, *Alp*, and *Col1a1* (Supplemental Figure 4C). These findings elucidated the critical role of PTH1R in determining the osteogenic capacity and differentiation process of OMSCs.

We performed tartrate-resistant acid phosphatase (TRAP) staining to further evaluate the effects of PTH1R depletion on bone resorption (Figure 2J). The number of TRAP^+^ osteoclasts was significantly elevated in PTH1R-cKO mice (Figure 2K). In addition, genes related to osteoclastogenesis and osteoclast maturation were statistically upregulated in alveolar bone from PTH1R-cKO mice, including cathepsin K (*Ctsk*), matrix metalloproteinase 9 (*Mmp9*), nuclear factor of activated T cells 1 (*Nfatc1*), ATPase H+ transporting V0 subunit d2 (*Atp6v0d2*), and tumor necrosis factor receptor superfamily 11a (*Tnfrsf11a*) (Figure 2L). We also assessed the bone resorption mediators secreted by osteoblasts. Significantly higher expression of tumor necrosis factor superfamily 11 (*Tnfsf11*) was detected in PTH1R-deficient mice and primary OMSCs accompanied by an unchanged *osteoprotegerin* (*Tnfrsf11b*) level, leading to higher *Tnfrsf11a*/*Tnfrsf11b* ratio (Figure 2L and Supplemental Figure 4D). These results indicated that bone formation and bone resorption activities were both enhanced upon PTH1R deletion in Gli1^+^ progenitors. The increase in bone resorption activity was greater than bone formation and ultimately led to decreased oral bone volume.

### PTH1R is a key regulator in inflammation-related oral bone diseases.

**Figure 3:**
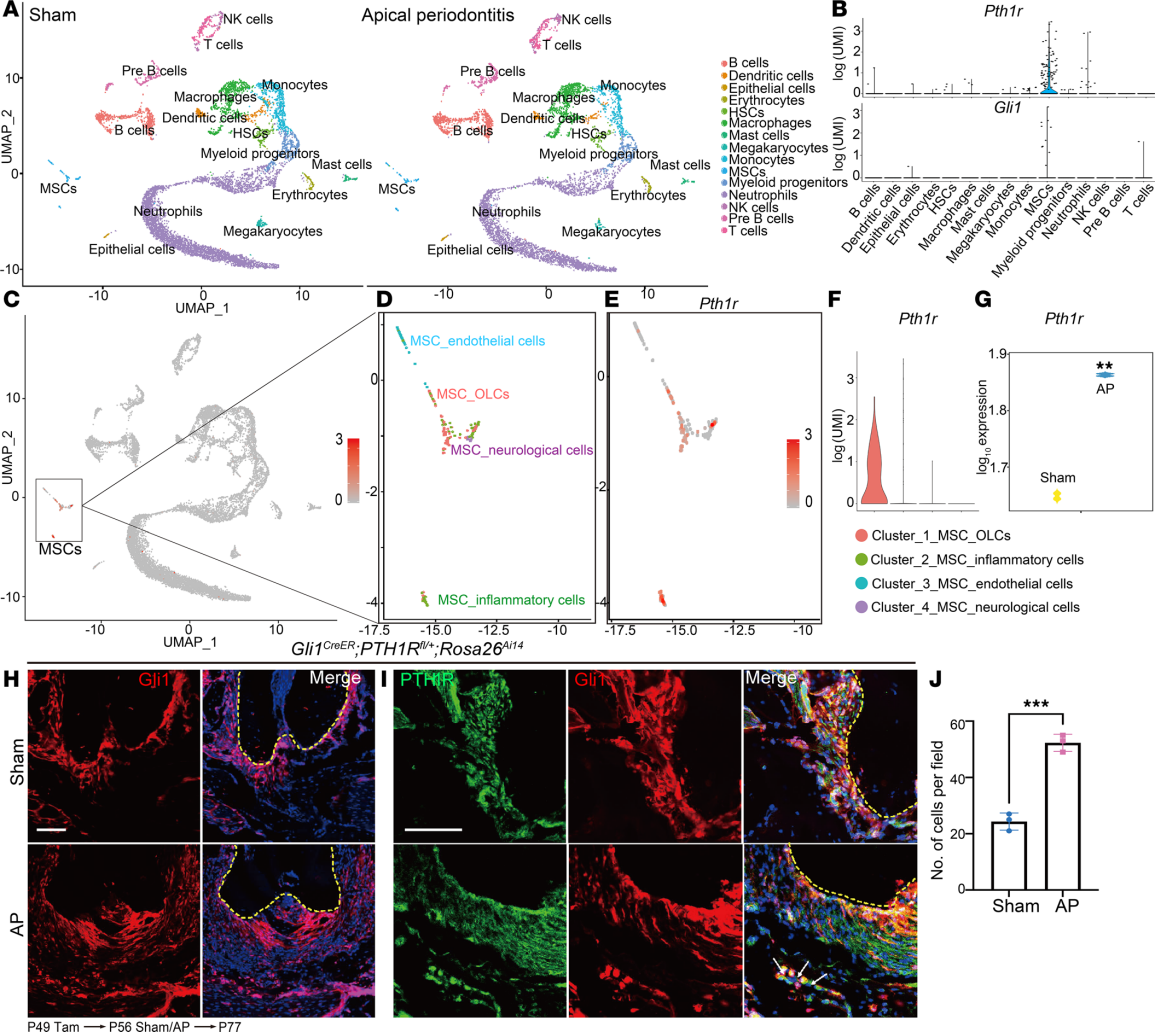
PTH1R is a key regulator in inflammation-related bone disease. (**A**) Uniform manifold approximation and projection (UMAP) visualization of aligned gene expression data showing 15 distinct clusters and cellular origin. The library consisted of cells extracted from mandibles of control mice (*n* = 8,340) and apical periodontitis (AP) mice (*n* = 6,808). (**B**) Violin plots of the expression of *Pth1r* and *Gli1* in all clusters. UMI, unique molecular identifier. (**C**) UMAP visualization of *Pth1r* in all clusters. (**D**) UMAP visualization of 4 MSC subclusters. The cell numbers in each MSC subcluster were as follows: control: 102 and AP: 129 for the MSC population, control: 42 and AP: 52 for MSC_OLCs, control: 30 and AP: 45 for MSC_inflammatory cells, control: 22 and AP: 29 for MSC_endothelial cells, control: 8 and AP: 3 for MSC_neurological cells. (**E**) The expression level of *Pth1r* in reclustered MSC population. (**F**) Violin plot of the expression of *Pth1r* in 4 MSC subclusters. (**G**) Violin plot of the expression of *Pth1r* in alveolar bone using bulk RNA-Seq analysis under control and AP conditions. (**H**) Lineage-tracing results of Gli1^+^ cells in control mice under both homeostasis and AP conditions at P77. Yellow dashed line indicates interface between tooth root and PDL. *n* = 3. (**I**) Immunofluorescence staining of PTH1R in healthy or inflammatory microenvironment of control mice. Yellow dashed line indicates interface between tooth root and PDL. White arrows depict PTH1R^+^Gli1^+^ cells. *n* = 3. (**J**) Quantification of PTH1R^+^Gli1^+^ cell numbers in periapical bone of *Gli1^CreER^ Rosa26^Ai14^* mice under homeostasis and AP conditions. *n* = 3. Scale bar = 100 μm. Data were all obtained in male mice. Data are mean ± SEM. **P < 0.01, ***P < 0.001.

PTH1R has a regulatory function in bone turnover, so we sought to further characterize its expression in oral bone based on a single-cell RNA sequencing (scRNA-Seq) analysis. We have established a library composed of cell populations from alveolar bone derived under both healthy and inflammatory conditions (23). Fifteen distinct cell clusters were identified, including hematopoietic stem cells (*Cd34*), MSCs (*Col1a1*), T cells (*Cd3g*), pre–B cells (*Vpreb1*), B cells (*Cd79a*), natural killer cells (*Klrd1*), dendritic cells (*Siglech*), myeloid progenitors (*Mpo*), neutrophils (*S100a8*), monocytes (*Ly6c2*), macrophages (*Adgre1*), mast cells (*Fcer1a*), megakaryocytes (*Gp1bb*), epithelial cells (*Epcam*), and erythrocytes (*Hbb-bt*) (Figure 3A). *Pth1r* and *Gli1* were predominately expressed in the MSC cluster (Figure 3B). The MSC population was reclustered into 4 subclusters: MSC_osteolineage cells (OLCs) (*Prrx1*, *Cxcl12*, *Runx2*, and *Sp7*), MSC_endothelial cells (*Cdh5*), MSC_inflammatory cells (*S100a8/S100a9*), and MSC_neurological cells (*Plp1*) (Figure 3, C and D). *Pth1r* was most highly expressed in MSC_OLCs (Figure 3, E and F), which was identified as the main participant in a protective role in inflammatory bone lesions (23). We therefore built an inflammation-related bone disease model by generating AP. Higher expression level of *Pth1r* in alveolar bone was detected in inflammatory conditions by RNA-Seq (Figure 3G). Gli1^+^ lineage cells were activated by inflammation and rapidly expanded toward the apical bone lesion (Figure 3H). Gli1^+^ lineage cells filled the inflammatory infiltration and centered on the apical foramen and oral bone. These cells were highly positive for PTH1R expression. We also observed higher PTH1R^+^Gli1^+^ cells embedded in the apical bone matrix adjacent to the infiltration area (Figure 3, I and J), implying PTH1R is an essential regulator during inflammation-related bone diseases.

### Gli1^CreER^ PTH1R^fl/fl^ mice exhibit restricted periapical lesion because of activated bone turnover.

**Figure 4:**
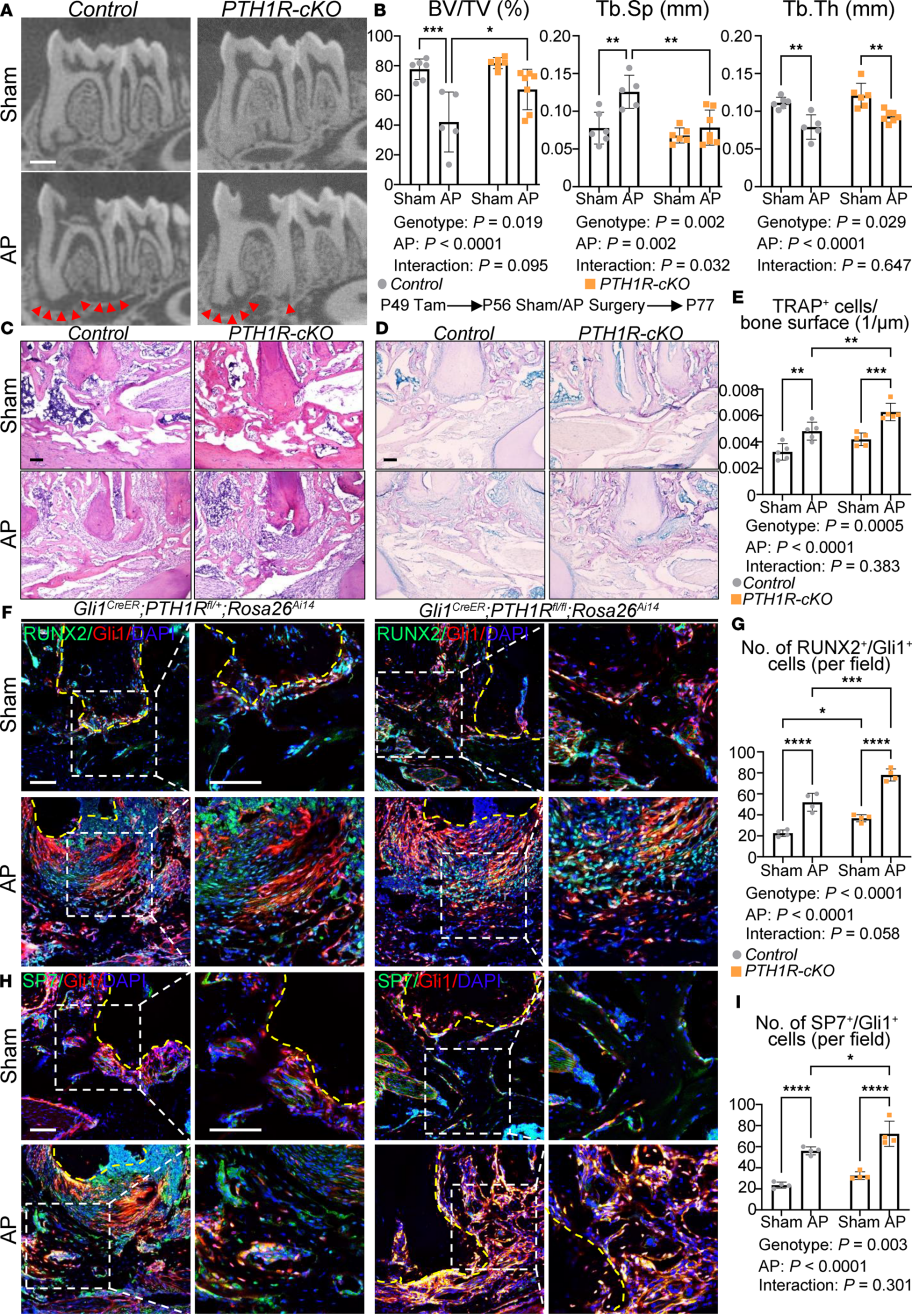
*Gli1^CreER^ PTH1R^fl/fl^* mice exhibit restricted periapical lesion because of activated bone turnover. (**A**) Two-dimensional micro-CT images of the mandibles from *Gli1^CreER^ PTH1R^fl/+^ Rosa26^Ai14^* and *Gli1^CreER^ PTH1R^fl/fl^ Rosa26^Ai14^* mice in sham and AP groups. Red arrowheads depict the region of periapical lesion. *n* = 6 for sham and *n* = 5 for AP of *Gli1^CreER^ PTH1R^fl/+^ Rosa26^Ai14^* mice. *n* = 6 for sham and *n* = 7 for AP of *Gli1^CreER^ PTH1R^fl/fl^ Rosa26^Ai14^* mice. (**B**) Quantitative analysis of trabecular bone parameters including BV/TV (%), Tb.Sp (mm), and Tb.Th (mm). (**C**) HE staining of the distal root of mandibular first molar showed the periapical lesion induced by AP. *n* = 3. (**D** and **E**) TRAP staining and quantification of TRAP^+^ cells/bone surface. *n* = 5. (**F** and **G**) Immunofluorescence staining and quantification showed increasing Runx2^+^Gli1^+^ cell numbers in periapical bone of *Gli1^CreER^ PTH1R^fl/fl^ Rosa26^Ai14^* mice under both homeostasis and AP conditions. Yellow dashed lines depict the region of distal root of the mandibular first molar. Boxed areas are shown at higher magnification. *n* = 4. (**H** and **I**) Immunofluorescence staining and quantification showed increased Sp7^+^Gli1^+^ cell numbers in inflammatory periapical bone of *Gli1^CreER^ PTH1R^fl/fl^ Rosa26^Ai14^* mice. Yellow dashed lines depict the region of distal root of the mandibular first molar. Boxed areas are shown at higher magnification. *n* = 4. Scale bar = 500 μm (**A**) and 100 μm (**C**, **D**, **F**, and **H**). Data were all obtained in male mice. Significance is determined using 2-way ANOVA with Tukey’s correction for multiple comparisons. Data are mean ± SEM. *P < 0.05, **P < 0.01, ***P < 0.001, ****P < 0.0001.

We next studied the specific function of PTH1R under conditions of inflammation. We challenged cohorts of mice with unilateral AP. As expected, following a 3-week period of inflammation, we observed significant reductions in BV/TV and Tb.Th accompanied by increased Tb.Sp in control oral bone in the presence of AP. Interestingly however, *Gli1^CreER^ PTH1R^fl/fl^ Rosa26^Ai14^* mice showed a smaller radiolucent area at the root furcation and apical region during inflammation. It was characterized by a significantly smaller periapical lesion area, higher BV/TV and Tb.Th, as well as lower Tb.Sp, when compared with the control littermates (Figure 4, A and B). Histological examination showed that AP led to inflammatory infiltration and activated bone resorption around the apex in both control and PTH1R-cKO mice (Figure 4C). Notably, PTH1R-cKO mice had different morphological trabecula structures during inflammation, accompanied by a higher number of TRAP^+^ osteoclasts (Figure 4, D and E). In addition, we found a few RUNX2^+^Gli1^+^ and SP7^+^Gli1^+^ cells present along the periapical bony surface in the homeostasis state (Figure 4, F–I). Notably, these cells were activated and recruited to the periapical lesion during inflammatory conditions, verifying the involvement of Gli1^+^ MSCs in the osteogenic process during inflammation (Figure 4, F and H). Interestingly, statistically higher numbers of RUNX2^+^Gli1^+^ and SP7^+^Gli1^+^ cells were detected in the apical lesions in mice lacking PTH1R (Figure 4, G and I), indicating that PTH1R deficiency in Gli1^+^ lineage cells accelerated osteoblast differentiation in the inflammatory microenvironment. This resulted in stronger protective activities and a restricted apical lesion area in PTH1R-cKO mice.

### PTH1R deletion upregulates IGF signaling under physiological and inflammatory conditions.

**Figure 5:**
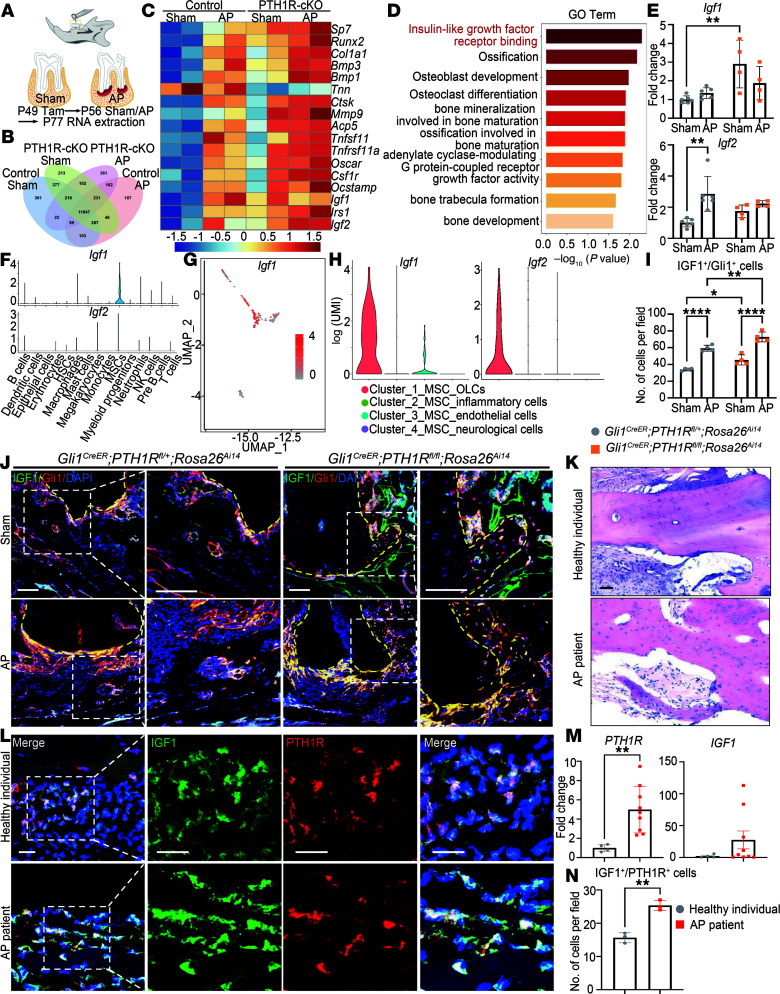
IGF1 plays an important role in regulating bone turnover under both homeostasis and inflammatory microenvironment. (**A**) Schematic diagram of the experimental design. (**B**) Venn diagram showing coexpressed genes among Control_Sham, Control_AP, PTH1R-cKO_Sham, PTH1R-cKO_AP (FPKM > 1). Male mice were used. FPKM, fragments per kilobase million. (**C**) Heatmap of representative genes associated with osteogenesis, osteoclastogenesis, and IGF signaling. *n* = 2 for each group. (**D**) GO analysis of Control_Sham versus PTH1R-cKO_Sham enriched GO terms related to insulin-like growth factor, bone formation, and bone resorption. (**E**) RT-qPCR of *Igf1* and *Igf2* expression in mandibles under control and inflammation. *n* = 6 for control and *n* = 4 for PTH1R-cKO. Male mice were used. (**F**) Violin plot of the expression of *Igf1* and *Igf2* in all clusters. (**G**) The expression level of *Igf1* in reclustered MSC population. (**H**) The expression level of *Igf1* and *Igf2* in 4 MSC subclusters presented in violin plot. (**I** and **J**) Immunofluorescence staining and quantification showed upregulated IGF1^+^Gli1^+^ cells in periapical bone of *Gli1^CreER^ PTH1R^fl/fl^ Rosa26^Ai14^* male mice under homeostasis and AP conditions. Yellow dashed lines depict the region of distal root of the mandibular first molar. Boxed areas are shown at higher magnification. *n* = 4. (**K**) HE staining of alveolar bone of healthy individuals and patients with AP. (**L** and **N**) Immunofluorescence double staining of PTH1R and IGF1 and quantification of IGF1^+^PTH1R^+^ in healthy and inflammatory alveolar bone of human samples. Boxed areas are shown at higher magnification. *n* = 3. (**M**) *PTH1R* and *IGF1* gene expression of human healthy and inflammatory alveolar bone tissues. *n* = 4 in healthy individuals and *n* = 9 in patients with AP. Scale bar = 100 μm (**J** and **K**), 25 μm (**L**). Significance is determined using unpaired 2-sided Student’s *t* tests between 2 groups and 2-way ANOVA with Tukey’s correction for multiple comparisons. Data are mean ± SEM. *P < 0.05, **P < 0.01, ****P < 0.0001.

We next performed RNA-Seq analysis on alveolar bone from control and mutant mice obtained under both physiological and inflammatory conditions (Figure 5A). We identified 668 genes that had significantly altered expression in PTH1R-cKO mice. Of these, expression of 296 genes was increased (Figure 5B). The gene expression pattern of regulators associated with osteogenesis (such as *Sp7*, *Runx2*, *Col1a1*, *Bmp1*, and *Tnn*) and osteoclastogenesis (such as *Ctsk*, *Mmp9*, *Acp5*, *Tnfrsf11a*, *Oscar*, and *Csf1r*) verified that PTH1R ablation in Gli1^+^ lineage cells activated bone remodeling (Figure 5C). Gene ontology (GO) analysis of upregulated genes revealed that pathways related to ossification, osteoblast differentiation, bone development, and osteoclast differentiation were activated. It is important to note that the upregulated genes in PTH1R-cKO that were enriched are in the insulin-like growth factor receptor binding pathway (Figure 5D). PTH1R deletion led to significantly elevated *Igf1* and *Igf2* expression. Kyoto Encyclopedia of Genes and Genomes (KEGG) analysis enriched PI3K-Akt signaling pathway, which was one of the major downstream pathways activated by IGF1-IGF1 receptor (IGF1R) (Supplemental Figure 5A). Gene set enrichment analysis revealed the PI3K-Akt signaling pathway was highly upregulated in PTH1R-cKO mice when compared with controls (Supplemental Figure 5B). Expression levels of *Igf*s were upregulated during AP, which remained higher in PTH1R-deficient mice in comparison with controls (Figure 5C). RT-qPCR further verified the expression pattern of *Igf*s (Figure 5E), implying the important function of IGF signaling after PTH1R deletion.

We also examined the expression pattern of *Igf*s at single-cell resolution. The results indicated that *Igf1* was enriched in the MSC cluster of alveolar bone, while *Igf2* expression was relatively lower in all clusters (Figure 5, F and H). We therefore focused on *Igf1* in the orofacial region. The violin plot indicated that, consistent with *Pth1r*, *Igf1* was abundantly expressed in MSC_OLCs. This indicates the potential interaction of PTH1R and IGF1 in the MSC cluster (Figure 5, G and H). We then characterized the IGF1 expression pattern in vivo by performing immunofluorescence staining. Under physiological conditions, IGF1 was found in osteoblasts, PDL cells and, to a lesser extent, in osteocytes (Figure 5J). As noted above, inflammation led to elevated IGF1 expression in osteoblasts and PDL surrounding the periapical lesion. Loss of PTH1R upregulated IGF1 production, as evidenced by higher numbers of IGF1^+^Gli1^+^ cells and *Igf1* transcripts (Figure 5, E, I, and J).

To understand the translational significance of our findings, we also collected human alveolar bone samples from healthy individuals and patients with AP to determine if IGF1 expression was altered during inflammation (Figure 5K). We observed a significant increase in *PTH1R* gene expression levels under inflammation while *IGF1* exhibited a trend toward upregulation in human alveolar bone under AP (Figure 5M). Furthermore, immunostaining showed a more extensive distribution of IGF1 in cells located in oral bone marrow, bone matrix, and bone lining cells and revealed a higher number of PTH1R^+^IGF1^+^ cells under inflammatory conditions (Figure 5, L and N), providing further support for the idea that IGF1 was upregulated in inflammatory bone diseases.

It is known that IGF1 stimulates radial bone growth and regulates bone properties via its effects on osteoblasts, osteocytes, and osteoclasts (24). Yet, the actions of IGF1 in periodontal tissue and oral bone remain under investigation. Therefore, we generated an IGF1 conditional knockout (IGF1-cKO) mouse model under control of Gli1 promoter. Micro-CT analysis revealed reduced alveolar bone mass in IGF1-cKO mice, characterized by significantly reduced BV/TV, Tb.Th, and Tb.Sp (Supplemental Figure 6A). HE staining verified a decrease in oral bone volume in IGF1-cKO mice (Supplemental Figure 6B). It was notable that loss of IGF1 in Gli1^+^ MSCs led to attenuated SP7 and RUNX2 expression, indicating decreased osteoblast activity and maturation (Supplemental Figure 6, D and E). Importantly, a reduction in osteoclast number was observed in IGF1-cKO mice compared with controls (Supplemental Figure 6C), implying a low bone turnover state in the absence of IGF1. These findings suggested that the low bone mass observed in IGF1-cKO mice was due to impaired bone remodeling, highlighting the crucial role of IGF1 in facilitating orofacial bone remodeling.

### Ablation of IGF1 ameliorates the aberrant bone remodeling in Gli1^CreER^ PTH1R^fl/fl^ mice.

**Figure 6:**
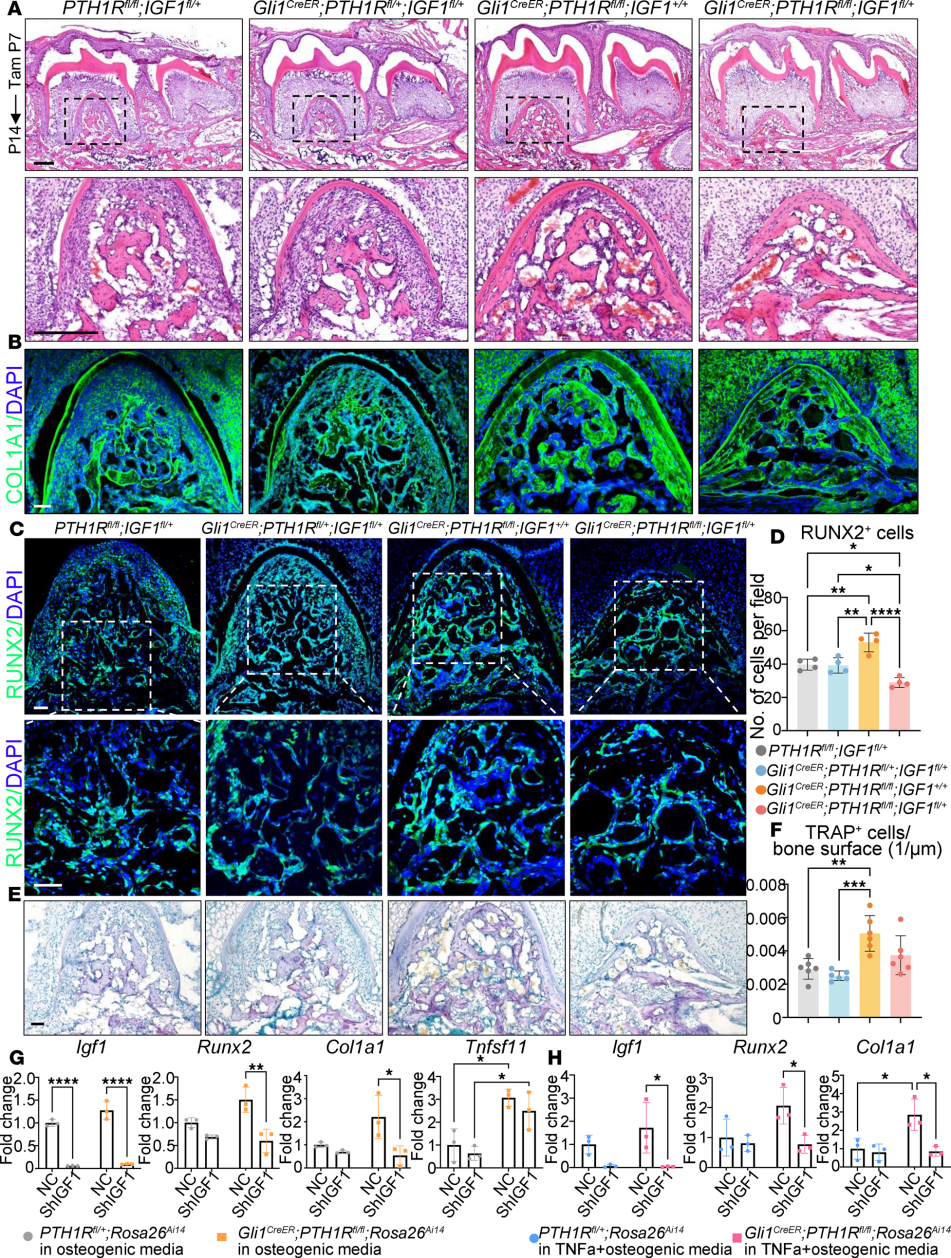
Lack of IGF1 in PTH1R-cKO mice reverses increased bone formation and bone resorption activities. (**A**) HE staining of control, *Gli1^CreER^ PTH1R^fl/+^ IGF1^fl/+^*, *Gli1^CreER^ PTH1R^fl/fl^ IGF1^+/+^*, and *Gli1^CreER^ PTH1R^fl/fl^ IGF1^fl/+^* mice at P14. Boxed areas are shown at higher magnification. *n* = 3. (**B**) Immunofluorescence staining of COL1A1 of each group. *n* = 3. (**C** and **D**) Immunofluorescence staining and quantification of Runx2 showed no difference between control and *Gli1^CreER^ PTH1R^fl/+^ IGF1^fl/+^* mice. PTH1R-cKO mice displayed increased Runx2^+^ cell number, the trend of which was reversed in *Gli1^CreER^ PTH1R^fl/fl^ IGF1^fl/+^* mice. Boxed areas are shown at higher magnification. *n* = 4. (**E** and **F**) TRAP staining and quantification exhibited no difference between control and *Gli1^CreER^ PTH1R^fl/+^ IGF1^fl/+^* mice. PTH1R-cKO mice had higher TRAP^+^ osteoclast numbers. The number of osteoclasts is in a downregulated trend in *Gli1^CreER^ PTH1R^fl/fl^ IGF1^fl/+^* mice compared with PTH1R-cKO mice. *n* = 6. (**G**) RT-qPCR results of *Igf1*, *Runx2*, *Col1a1*, and *Tnfsf11* expression in control and IGF1-knockdown OMSCs cultured in osteogenic media. *n* = 3. (**H**) RT-qPCR results of *Igf1*, *Runx2*, and *Col1a1* expression in control and IGF1-knockdown OMSCs cultured in TNF-α (10 ng/mL, MilliporeSigma) added to osteogenic media. *n* = 3. Scale bar = 200 μm (**A**), 50 μm (**B**, **C**, and **E**). Male mice were used. Significance is determined using 1-way ANOVA with Tukey’s correction in **D** and **F** and 2-way ANOVA with Tukey’s correction for multiple comparisons in **G** and **H**. Data are mean ± SEM. *P < 0.05, **P < 0.01, ***P < 0.001, ****P < 0.0001.

As noted above, IGF1 expression was significantly upregulated after PTH1R ablation. We wanted to understand whether the higher IGF1 was responsible for the aberrant bone remodeling observed in PTH1R-cKO mice. We subsequently ablated both IGF1 and PTH1R in Gli1^+^ mesenchymal progenitors. *Gli1^CreER^ PTH1R^fl/+^ IGF1^fl/+^* mice showed unchanged growth of oral bone and periodontal tissues, as evidenced by comparable alveolar bone volume and well-organized PDL structure compared with control littermates. Dual deletion of PTH1R and IGF1 resulted in fewer bony structures at the furcation area of mandibular bone, accompanied by impaired PDL organization and truncated dental root (Figure 6A). We observed significantly reduced IGF1 expression in alveolar bone of *Gli1^CreER^ PTH1R^fl/fl^ IGF1^fl/+^* mice (Supplemental Figure 7). Immunofluorescence staining showed that Runx2 and Col1a1 expressions were significantly increased in PTH1R-cKO mice. This phenotype was rescued in conjunction with heterozygous IGF1 deletion (Figure 6, B–D). Furthermore, the higher bone resorption observed in PTH1R-deficient mice was corrected in the double mutants, as evidenced by reduced TRAP^+^ osteoclasts when compared with PTH1R-cKO mice (Figure 6, E and F). We subsequently performed cellular experiments to provide in vitro evidence. The results showed that under both normal and inflammatory conditions, knockdown of IGF1 by shRNAs led to significantly reduced *Runx2* and *Col1a1* expression in OMSCs, which was more significantly evident in OMSCs that lack PTH1R. A trend toward lower *Tnfsf11* expression was observed with *Igf1* knockdown (Figure 6, G and H). These data further verified that IGF1 is a major downstream factor in PTH1R-cKO mice driving the changes in craniofacial bone remodeling.

### Activated Hedgehog signaling contributes to the elevated IGF1 in PTH1R-cKO mice.

**Figure 7:**
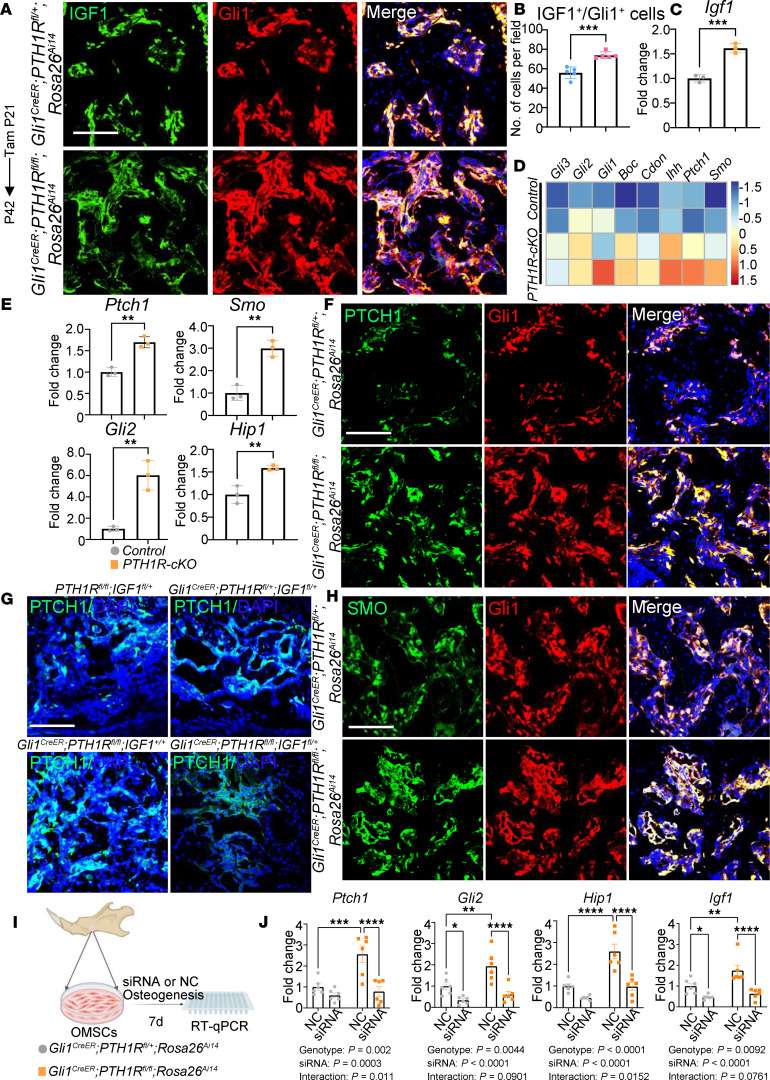
Activated Hedgehog signaling contributes to the increased IGF1 in PTH1R-cKO mice. (**A** and **B**) Immunofluorescence staining and quantification showed increased IGF1^+^Gli1^+^ cell number in root furcation area of *Gli1^CreER^ PTH1R^fl/fl^ Rosa26^Ai14^* female mice at P42. *n* = 5. (**C**) RT-qPCR results showed upregulated *Igf1* in PTH1R-cKO OMSCs. *n* = 3. (**D**) Heatmap depicting the expression of Hh signaling–related genes analyzed by RNA-Seq in control and PTH1R-cKO alveolar bone samples. *n* = 2. (**E**) RT-qPCR results showed upregulated Hh signaling–related genes (*Ptch1*, *Smo*, *Gli2*, *Hip1*) in PTH1R-cKO OMSCs. *n* = 3. (**F** and **H**) Immunofluorescence staining of Ptch1 and Smo in Gli1^+^ progenitors of *Gli1^CreER^ PTH1R^fl/+^ Rosa26^Ai14^* and *Gli1^CreER^ PTH1R^fl/fl^ Rosa26^Ai14^* mice at P42. *n* = 3–5. (**G**) Immunofluorescence staining of Ptch1 showed no difference between control and *Gli1^CreER^ PTH1R^fl/+^ IGF1^fl/+^* mice. PTH1R-cKO mice had activated Ptch1 expression, which was downregulated in *Gli1^CreER^ PTH1R^fl/fl^ IGF1^fl/+^* compared with PTH1R-cKO mice. *n* = 3. (**I**) Schematic representation of the experimental design for cellular studies using siRNA. (**J**) Gene expression profile of Hh signaling–related markers and *Igf1* in OMSCs of control and PTH1R-cKO after siRNA treatment. *n* = 6. Scale bar = 100 μm. Significance is determined using unpaired 2-sided Student’s *t* tests between 2 groups and 2-way ANOVA with Tukey’s correction for multiple comparisons. Data are mean ± SEM. *P < 0.05, **P < 0.01, ***P < 0.001, ****P < 0.0001.

It is well established that PTH can stimulate IGF1 synthesis via a cAMP-dependent mechanism (25–27). Mouse data and clinical studies also suggest that the anabolic effects of PTH are partially modulated by IGF1 (24). We also observed upregulation of *Igf1* transcripts upon PTH (1-34) and an adenylate cyclase activator, forskolin, administration in cultured cells (Supplemental Figure 8A), verifying that the synthesis of *Igf1* is one of the major effects of PTH/cAMP signaling. Interestingly, however, we observed upregulated *Igf1* and *Igf2* expression in mice that lack PTH1R (Figure 7, A–C, and Supplemental Figure 9, A–C). We therefore hypothesized that an alternative signaling pathway may drive the higher IGF levels observed in PTH1R-cKO mice. A previous study uncovered a positive feedback mechanism between Hedgehog (Hh) and Igf signaling during osteoblast differentiation. In particular, Hh signaling induces transcription of multiple members of the Igf family (28). We hypothesized that activation of Hh signaling in PTH1R-cKO mice was responsible for the upregulated Igf signaling. Indeed, we detected higher expression of Hh signaling target genes in PTH1R-cKO mice by RNA-Seq (Figure 7D). RT-qPCR results further verified upregulated patched 1 (*Ptch1*), Smoothened (*Smo*), *Gli2*, and huntingtin interacting protein 1 (*Hip1*) in PTH1R-deficient OMSCs (Figure 7E). Ptch1 is a transcriptional target of Hh signaling (29), and Gli2 is the major Gli transcription factor that activates downstream target gene expression in Hh signaling (30). As an in vivo readout of Hh signaling activity, we found Ptch1 expression was stronger in PTH1R-deficient mice (Figure 7F), which was corrected by dual ablation of IGF1 and PTH1R (Figure 7G), indicating the activation of Hh signaling upon PTH1R deletion. Moreover, the Hh receptor Smo (31), which is required to transduce the signaling from Hh ligands, was significantly upregulated in PTH1R-cKO mice (Figure 7, D and E). Furthermore, we found enhanced Smo expression levels, which correlated with Gli1^+^ MSCs in PTH1R-cKO by immunofluorescence staining (Figure 7H).

To mechanistically investigate whether the higher IGF1 levels in Gli1^+^ progenitors was due to Hh activation, we applied siRNA to silence Gli1/2 in order to directly evaluate the changes in IGF1 levels in response to PTH1R deletion in OMSCs (Figure 7I). Our findings revealed that knockdown of Gli1/2, as the primary effector for Hh-mediated transcriptional activation, not only attenuated *Ptch1*, *Gli2*, and *Hip1* induction but also reduced the expression of *Igf1* in OMSCs (Figure 7J). Moreover, we applied Smoothened Agonist (SAG), a Smo receptor agonist that activates the Hh signaling pathway in OMSCs. Our results demonstrated that SAG significantly induced mRNA levels of Hh transcriptional targets, such as *Ptch1*, *Gli2*, and *Hip1*, at 48 hours posttreatment. Importantly, SAG also activated expression of *Igf1* in OMSCs (Supplemental Figure 9D), underscoring the involvement of Hh signaling in mediating increased IGF1 levels due to PTH1R deletion in Gli1^+^ progenitors. Taken together, these data indicated that loss of PTH1R signaling activated Hh signaling in orofacial tissues, and this may contribute to higher IGF1 expression, which in turn stimulates bone formation and resorption.

## Discussion

We have identified pivotal mechanisms by which PTH1R signaling regulates intramembranous ossification and periodontium development. Loss of PTH1R in Gli1^+^ MSCs led to activation of Hedgehog signaling, which upregulated *Igf1* expression in the orofacial tissues. IGF1 drives both osteoblast and osteoclast differentiation but appears to have a greater effect on osteoclasts. This imbalance subsequently led to reduced orofacial bone volume and defects in the PDL. Periodontal tissues, including alveolar bone, PDL, and cementum, share the same embryonic origin and are all derived from cranial neural crest cells (32). There are diverse stem and progenitor cells residing in the craniofacial region, which participate in the formation, maintenance, and regeneration of orofacial tissues (33). The unique embryonic origin and mode of intramembranous ossification make it necessary to elucidate the specific mechanisms of MSC fate decision, bone remodeling, and repair. This will enable innovations in therapeutic strategies for craniofacial osseous defects and inflammatory-related bone diseases.

The PTH1R signaling pathway is essential to bone homeostasis. Proper cell fates of mesenchymal progenitor cells are also tightly maintained by PTH1R signaling in various tissues (21, 34, 35). Human mutations of PTH1R result in multiple disorders in the craniofacial region. Clinical evaluation of human fetuses affected with embryonic lethal Blomstrand-type chondrodysplasia caused by homozygous loss-of-function mutations in PTH1R exhibit severe alveolar bone distortion (36). Primary failure of tooth eruption caused by heterozygous PTH1R mutations resulted in growth deficiency of the alveolar process in the affected region (37, 38). Furthermore, movement of the teeth by orthodontic treatment leads to fusion of dental cement with surrounding bone (39). The role of PTH1R has been identified in various stem cell populations, including Prrx1^+^, Lepr^+^, Sp7^+^, and PTHrP^+^ lineage cells. PTH1R exerts diverse regulatory functions on different lineages of mesenchymal progenitors. Global deletion of PTH1R resulted in excessive mineralization and a synchondrosis of the skull (20). Deletion of PTH1R in Sp7-expressing progenitors led to disorders in dental root development, failure of tooth eruption, and accelerated cementoblast differentiation (19). Similarly, ablation of PTH1R in PTHrP^+^ dental follicle progenitor cells (DFPCs) resulted in loss of functionality in the periodontal attachment apparatus. PTH1R-deficient DFPCs had upregulated bone/cementum matrix protein and subsequently generated nonphysiological cementoblast-like cells (9). In contrast with this enhanced mineralization, our previous study specifically ablated PTH1R in Prrx1^+^ progenitors and found reduced osteoblast differentiation in the alveolar bone, leading to arrested tooth eruption (12). Similar results were observed in mice lacking PTH1R in Lepr^+^ mesenchymal progenitors, where significantly decreased bone formation rate and bone mineral density were detected. Lack of PTH1R in Lepr^+^ cells also impaired the oral bone repair after injury (8). These studies highlight the prominent role of PTH1R in craniofacial development and growth. However, the regulatory function of PTH1R in various stem cells has been controversial, and the underlying mechanisms needed to be further elucidated. Our lineage-tracing experiments observed that Gli1^+^ cells could give rise to PDL cells, cementoblasts, osteoblasts, and osteocytes. This is consistent with the idea that Gli1 is a critical marker for periodontium tissue (11). Thus, we generated mice with a targeted deletion of PTH1R in Gli1^+^ mesenchymal progenitors to understand the molecular mechanisms that specifically act in the periodontium tissue. Our findings in this study imply that PTH1R signaling supports periodontium development and negatively controls intramembranous bone formation. Mice that lack PTH1R in Gli1^+^ lineage cells exhibited increased bone remodeling and disruption in PDL formation. PTH1R-cKO mice exhibited accelerated bone formation rate and osteogenic differentiation in vivo. PTH1R-deficient OMSCs also had enhanced mineralization. These results suggested PTH1R suppresses the differentiation of Gli1^+^ progenitors toward osteoblasts in the craniofacial region. Micro-CT analysis showed a marked difference in Tb.N in male PTH1R-cKO mice but not in females, despite an increase in Tb.Sp being evident in both sexes. It was notable that sex differences were observed in the trabecular microarchitecture of the distal femur metaphysis in long bones, which showed higher Tb.N in males than females (40). Further analysis is required to determine whether sexual dimorphism in alveolar trabecular bone also exists.

PTH and PTHrP both signal through PTH1R (16). PTH exerts its biological function in various mesenchymal lineages in the craniofacial region, including OMSCs, dental pulp stem cells, PDL stem cells, stem cells from apical papilla, and tooth germ progenitor cell (8, 12, 19, 41–43). PTH is generally considered as a positive regulator of stem/progenitor cell fate in the orofacial region. PTHrP is a locally acting autocrine/paracrine ligand that exerts pleiotropic effects on cell proliferation and differentiation during embryonic skeleton development and postnatal bone formation. PTHrP is specifically expressed in cells of mesenchymal lineage in dental follicle (DF) or in odontoblasts. PTHrP^+^ DF cells are able to differentiate into PDL fibroblasts, cementoblasts, and osteoblasts of alveolar bone (9). We hypothesized the ligand responsible for the observed phenotypes is predominantly PTHrP, since it is expressed in dental mesenchymal lineages such as Gli1^+^ and Sp7^+^ progenitors. PTH1R deficiency affected the PTHrP-PTH1R autocrine regulation of Gli1^+^ mesenchymal progenitor cells, leading to a shift in physiological cell fates and accelerated differentiation toward osteoblasts, consistent with the observation of premature synchondrosis closure in these mutants (44). The contrasting functions of PTH1R in modulating skeletal mineralization is possibly due to the spatial and temporal onset of *Cre* expression in diverse stem cell populations and the distinct role of PTH and PTHrP.

In addition to regulating craniofacial tissue growth under homeostasis, we also identified the pivotal role of the PTH1R signaling pathway under inflammation-related bone disease conditions. Patients with hyperparathyroidism experience loss of lamina dura, reduced cortical bone thickness, and osteolytic lesions in the orofacial region. Additionally, there were increased signs of bone loss under periodontitis in the hyperparathyroidism group (45). In the current study, we noted that *Pth1r* was predominantly expressed in the MSC cluster during inflammation at single-cell resolution. The MSC cluster formed an increased self-supporting network by inducing osteogenesis and interacting with immune cells during inflammation (23). Loss of PTH1R led to reduced periapical lesions, which was likely attributed to the accelerated osteogenic potential of Gli1^+^ cells. We detected upregulated osteogenesis-related markers by RNA-Seq in PTH1R-cKO mice during inflammation. Corresponding with this observation, increased numbers of RUNX2^+^Gli1^+^ and SP7^+^Gli1^+^ cells were detected in the cryptal bone surrounding the periapical lesion. However, this finding is strikingly inconsistent with the clinical outcome of PTH or PTHrP treatment. Intermittent administration of teriparatide, an anabolic agent for osteoporosis approved by the Food and Drug Administration, achieved significant bone gain in periodontal surgery (46). Animal studies also suggested that intermittent PTH treatment could prevent alveolar bone destruction with AP-associated bone loss (47–49). Furthermore, PTHrP was found to be positive in the vascular zone, the pulp stroma, as well as the odontoblastic and subodontoblastic zones of inflamed dental pulp (50). It is reported that low, medium, and high doses of PTHrP (1-34) could prevent upregulation of IL-1β and IL-6 secretion and in turn inhibit alveolar bone loss in diabetic rats (51). These seemingly conflicting results imply that PTH1R signaling functions could be temporally distinct and are altered during inflammation. We hypothesized that although the protective function of PTH or PTHrP was diminished in PTH1R-cKO under AP, PTH1R ablation led to upregulated IGF1, which was mainly responsible for the upregulated osteogenesis and osteoclastogenesis. The augmentation of osteogenic characteristics of PTH1R-deficient progenitors in alveolar bone marrow contributed to the restricted alveolar bone loss under inflammation. In human patients with AP, we observed higher IGF1 expression in alveolar bone, implying that IGF1 may play a crucial role in regulating the inflammatory response in alveolar bone during AP. Further investigation is required to elucidate the specific mechanisms and determine whether IGF1 could serve as a potential therapeutic target for managing inflammatory dental diseases.

An important finding from our work concerned the upregulation of IGF1 in mice deficient in the PTH1R. IGF1 is a key regulator of tissue growth and development (24). It is expressed in bone marrow stromal cells, osteoblasts, and chondrocytes and facilitates skeletal development and regeneration (52–54). In long bones, IGF1 promotes osteoblastogenesis and prohibits osteoblast apoptosis (55). But it also induces Tnfsf11 synthesis and subsequently stimulates osteoclastogenesis, both indirectly and through direct activation of the IGF1R on osteoclasts (56). IGF1^–/–^ and IGF1R^–/–^ mice displayed delays in embryonic ossification of the cranial and facial bones (57). In contrast, IGF1 overexpression in late-differentiated osteoblasts resulted in thickening of calvaria bone (58). We generated conditional IGF1-knockout mice to further evaluate the site-specific function of IGF1. We found that depletion of IGF1 in Gli1^+^ progenitors resulted in significantly reduced osteoblast differentiation and maturation, accompanied by markedly suppressed bone resorption. This supports a positive role for IGF1 in regulating oral bone turnover. Thus, we speculated that the higher IGF1 detected in PTH1R-cKO mice was responsible for the active bone remodeling. Indeed, ablation of IGF1 ameliorated the aberrant higher osteogenic markers in PTH1R-deficient mice and primary OMSCs. It is noteworthy that IGF1 has been demonstrated to have an effect on bone resorption in vivo, which may compromise its positive effect on bone formation and limits its potential as an anabolic agent (58, 59). But those findings fit the current paradigm that PTH1R-cKO mice had higher bone resorption that exceeded bone formation, resulting in reduced oral bone volume.

There is substantial evidence that IGF1 serves as an essential mediator of PTH activity. Depletion of IGF1, IGF1R, and insulin receptor substrate 1 blocked the response to intermittent PTH treatment (60–62). Furthermore, teriparatide treatment of premenopausal women caused upregulation of IGF1R expression in circulating osteoblast progenitors, which correlated directly with bone mineral density (63). These animal and clinical studies imply that the anabolic effects of PTH are partially dependent on tissue IGF1. It was notable that heterozygous deletion of IGF1 did not result in impaired oral bone and PDL development. The effect of IGF1 became evident in conjunction with PTH1R ablation in the IGF/PTH1R dual-deletion mouse model and RNAi experiments, suggesting the action of IGF1 on osteogenesis relies on PTH1R signaling. These data reveal a negative feedback mechanism whereby PTH1R deletion upregulates IGF1 to compensate, implying a regulatory pathway between PTH1R and IGF1.

It is well known that PTH stimulates IGF1 expression (64). But surprisingly, we observed upregulated IGF1 production upon PTH1R ablation. We speculate that the higher IGF1 level was due to activation of Hh signaling. Several lines of evidence support that tenet. For example, we found higher expression of Hh signaling target genes *Ptch1*, *Gli2*, and *Hip1* in PTH1R-deficient mice. Moreover, in vivo PTCH1 was activated in PTH1R-cKO, and additional ablation of IGF1 corrected this condition. It has also been shown that Hh and Igf signaling exert synergistic interactions during tissue development. Hh signaling regulates IGF1 and IGF2 production in osteoblasts in a positive feedback loop (28). We also noted upregulated IGF1 at transcript and protein levels in Gli1^+^ cells that lack PTH1R, which was reversed by knockdown of Gli1/2. Depletion of Gli2 in vivo to rescue the elevated IGF1 level in PTH1R-cKO would provide more evidence, which is a limitation of the current study. Furthermore, IGFs can directly upregulate several of the IGF binding proteins, particularly insulin-like growth factor binding protein 3 (IGFBP3) and IGFBP6, possibly as a means to facilitate transport to the IGF1R or to distinct IGFBP receptors (65, 66). In our study, Both IGFBP3 and IGFBP6 were found to be markedly enhanced by RT-qPCR (Supplemental Figure 8B), and IGFBP receptor binding was the top GO term in PTH1R-cKO mice. Furthermore, Hh signaling triggers the relocation of the 7-pass transmembrane protein Smo, resulting in activation of downstream cellular events (67). Genetic studies of Smo in the mouse liver have also demonstrated Hh signaling can upregulate IGF1 (68). Smo expression was elevated in PTH1R-deficient Gli1^+^ MSCs, in accordance with the increased IGF1 expression. Application of SAG significantly increased *Igf1* in OMSCs. Finally, it is of note that Xu et al. reported that Gα_s_ inhibits Hh signaling activity during cranial bone development. Gα_s_ is a subunit of Gs that stimulates the cAMP-dependent pathway by activating adenyl cyclase. PTH1R is predominantly coupled to Gα_s_ (16, 69), and PTH1R may be one of the GPCRs that modulates Gα_s_ signaling during craniofacial development. Also, previous studies noted that Gα_s_ has a negative effect on Hh signaling downstream action of Smo by inhibiting Gli activities during ectopic bone formation (70). It is likely that the function of PTH1R/Gα_s_ signaling in ectopic bone formation reflects a role in directing intramembranous ossification by suppressing Hh signaling. It is conceivable that PTH1R inhibits orofacial ossification by suppressing Hh signaling and IGF1 production during craniofacial bone development and remodeling.

Taken together, our study demonstrated that Gli1^+^ MSCs are important stem cells responsible for craniofacial tissue development and formation. PTH1R signaling in Gli1^+^ cells controls oral bone remodeling and PDL turnover under homeostatic and pathological conditions. PTH1R couples ossification and bone resorption by negatively regulating Hh signaling and IGF1 production. These data expand previous knowledge of PTH1R and IGF1 signal transduction in MSCs’ differentiation and may provide insights into disease diagnosis and development of treatments for craniofacial diseases and inflammation-related bone disorders.

## Methods

### Sex as a biological variable.

Our study examined male and female animals and patients. For each type of experiments, we used the same sex as control to exclude the bias resulting from sex. Similar findings were reported for both sexes. Sex was not considered as a biological variable.

### Animals.

*Gt(ROSA)26Sor^tm14(CAG-tdTomato)Hze^* mice (*Rosa26^Ai14^*) (catalog JAX:007914), *Gli1^CreER^* (catalog JAX:007913) mice, and *IGF1^fl/fl^* mice (catalog JAX:012663) were purchased from The Jackson Laboratory. *PTH1R^fl/fl^* mice were obtained from Henry Kronenberg (Massachusetts General Hospital, Boston, Massachusetts, USA) and described previously (12, 71). By crossing *Gli1^CreER^ Rosa26^Ai14^* mice with *PTH1R^fl/fl^* mice, we attained the first generation of heterozygous *Gli1^CreER^ PTH1R^fl/+^ Rosa26^Ai14^*. The heterozygous mice were mated with *PTH1R^fl/fl^* mice to generate *Gli1^CreER^ PTH1R^fl/fl^ Rosa26^Ai14^* (PTH1R-cKO) mice. A similar strategy was used to breed *Gli1^CreER^ IGF1^fl/fl^* and *Gli1^CreER^ PTH1R^fl/fl^ IGF1^fl/+^* mice. All mice involved in this study were genotyped, the primer sequences of which are listed in the Supplemental Table 1. Mice at P14, P21, P49, or P84 were injected intraperitoneally with tamoxifen (MilliporeSigma) at a dosage of 2.5 mg/10 g body weight every 2 days for 3 times. Tamoxifen at a dosage of 0.1 mg/g was injected to the mice at P7 intraperitoneally once. All animal experiments were performed in accordance with the standards of the Institutional Animal Care and Use Committee at the State Key Laboratory of Oral Diseases, Sichuan University (WCHSIRB-D-2021-339).

### Mouse perfusion and sample harvest.

Mice were sacrificed by cervical dislocation, and then 10 mL 4% paraformaldehyde (PFA) was infused transcardially for perfusion. Mandibles were dissected and fixed in 4% PFA overnight and then stored in PBS at 4°C before processing.

### Micro-CT analysis.

The samples were scanned using a μCT50 scanner (Scanco), with a resolution of 7.0 μm per pixel. Regions of interest (ROIs) in normal alveolar bone were selected from the root furcation of the mandibular first molars. In the horizontal plane, the bone area between medial and distal root apex was used to set ROIs in AP and sham models. Bone-related parameters were measured to analyze alveolar bone features and AP lesions (72).

### Histology and immunostaining.

After decalcification using 20% EDTA (pH 7.5), samples were embedded in Tissue-Tek O.C.T. Compound (Sakura) and cut into 8 μm sections using CryoStar NX50 (Thermo Fisher Scientific). The sections were stained with hematoxylin (Biosharp) and eosin (Solarbio). TRAP (MilliporeSigma) staining was performed according to the manufacturer’s protocol. For immunostaining, slides were incubated with primary antibody overnight at 4°C, then incubated with Alexa Fluor 488 (1:1,000, Invitrogen, A11070) for 1 hour at room temperature. The primary antibodies included anti-RUNX2 (1:200, Abcam, ab23981), anti-SP7 (1:200, Abcam, ab22552), anti-COL1A1 (1:200, Abcam, ab21286), anti-PERIOSTIN (1:200, Abcam, ab14041), anti-IGF1 (1:50, R&D Systems, Bio-Techne, AF791), anti-IGF1 (1:50, Santa Cruz Biotechnology, sc518040), anti-IGF2 (1:200, Abcam, ab9574), anti-PTCH1 (1:100, ABclonal, A14772), anti-SMO (1:50, Santa Cruz Biotechnology, sc166685), and anti-PTH1R (1:200, Assay Biotech, G220). DAPI (Vector Labs, H1200) was used for nuclei counterstaining. Immunostaining images were captured using an Olympus confocal microscope FV3000. The counting of fluorescence images was performed using ImageJ software (NIH). The double-positive cells in a microscopic field of each section were counted. At least 6 different sections were used from each sample, and 3 or more different biological samples were analyzed for each group.

### Bone histomorphometry analysis.

Calcein double labeling was performed to evaluate dynamic mineral apposition (73). Mice were injected with 20 mg/kg of calcein (MilliporeSigma) at 6 and 2 days prior to sacrifice at 6 weeks old. We have also performed the bone histomorphometry analysis in older mice by injection with calcein at 8 and 2 days prior to sacrifice around 16 weeks old. Tamoxifen was injected around 12 weeks of age. Undecalcified mandibles were processed using a Multipurpose Cryosection Preparation kit (Section-LAB Co. Ltd) to obtain 8 μm sections. Parameters including MS/BS, MAR, and BFR/BV were measured by the Osteometrics software (Decatur).

### AP mouse model.

The unilateral AP model was generated using a dental handpiece to expose the pulp chambers of the mandibular first molar for 3 weeks (23, 74). Mice at approximately 8 weeks of age were used for the surgical procedures. Although this age group is commonly employed in establishing the model, their relatively young age may present a limitation in the context of animal experimentation.

### ScRNA-Seq library preparation and sequencing.

ScRNA-Seq libraries were built and preprocessed as described previously (23). Briefly, 20 C57BL/6 (GemPharmatech Co., Ltd.) male mice in each group were utilized to obtain single-cell suspensions. Mandibles were dissected, with soft tissues, molars, incisors, and bone from behind the condyle being removed. Subsequently, alveolar bone tissue was digested with 3 mg/mL collagenase type I (Gibco) and 4 mg/mL dispase II (MilliporeSigma) for 60 minutes at 37°C. Transcriptome libraries for single cells were captured using Chromium Controller (10x Genomics). RNA from the barcoded cells was subsequently reverse-transcribed, and sequencing libraries were constructed utilizing reagents from a Chromium Single Cell 3′ v3 reagent kit (10x Genomics), followed by sequencing on the NovaSeq system (Illumina). We used fastp to perform basic statistics on the quality of the raw reads. We utilized Cell Ranger (v3.1, 10x Genomics) for alignment of reads to the mouse genome mm10 and for cell detection using default parameters. Subsequently, we employed the Seurat package (v3.1) for analysis of the scRNA-Seq data. A gene with expression in more than 3 cells was considered expressed, and each cell was required to have at least 200 expressed genes. Cells with erythrocyte gene expression higher than 5% were filtered out. Based on filtered gene expression matrix by Seurat, differential expression analysis between samples was conducted using the edgeR package. We used clusterProfiler R package to test the statistical enrichment of marker genes in KEGG pathways.

### Bulk RNA-Seq analysis.

Sham and AP mandibles collected from control and PTH1R-cKO mice were used to extract total RNA using PowerLyzer 24 Homogenizer (QIAGEN) and TRIzol (Invitrogen) according to the manufacturer’s protocol. NanoDrop ND-1000 (Thermo Fisher Scientific) was used to quantify RNA concentration. Bulk RNA-Seq libraries were generated using the NEBNext Ultra RNA Library Prep Kit for Illumina (New England Biolabs), and index codes were added to correlate sequences to each sample. The library preparations were sequenced on an Illumina NovaSeq 6000 (LC-Bio Technology Co., Ltd.). The sequence quality was verified using fastp. StringTie was used to perform expression level for mRNAs by calculating FPKM. The differentially expressed mRNAs were selected with fold-change > 2 or fold-change < 0.5 and with parametric *F* test comparing nested linear models (*P* < 0.05) by edgeR. Genes were subjected to GO and KEGG enrichment analysis using the Database for Annotation, Visualization, and Integrated Discovery.

### RT-qPCR.

A PrimeScript RT reagent kit (Takara) was used to perform the reverse transcription. SYBR Green Supermix (Bio-Rad Laboratories) was used for RT-qPCR according to the manufacturer’s protocol. The relative expression was calculated using 2^ΔΔCT^ method with β*-actin* in mice and GAPDH in human samples as the internal control. All primers were listed in the Supplemental Table 2.

### Cell culture.

Primary OMSCs were digested from male mandibles using dispase II (4 mg/mL, MilliporeSigma) and collagenase type I (3 mg/mL, Gibco) for 1 hour. The control OMSCs were extracted from *PTH1R^fl/fl^ Rosa26^Ai14^* mice at P42. The PTH1R-cKO OMSCs were isolated from PTH1R-cKO mice, which received tamoxifen injection at P21, then were sacrificed at P42. Cell culture was performed in α-MEM (Gibco) supplemented with 10% fetal bovine serum (Gibco) and 1% penicillin-streptomycin (HyClone) at 37°C, 5% (v/v) CO_2_. OMSCs were used for experiments at passage 3–4 and plated in 12-well plates for mRNA extraction and 48-well plates for staining. For osteogenic induction, 10 mmol/L β-glycerophosphate and 50 μg/mL ascorbic acid were added to the culture medium and changed every 2 days. ALP staining (Beyotime) was performed after osteogenic induction for 7 days and ARS staining (MilliporeSigma) for 14 days according to the manufacturer’s instructions. For activation of Hh signaling pathway, OMSCs were treated with SAG (Yeasen) (300 nM) for 48 hours.

### Transfection.

For shRNA transfection, *Igf1* shRNA–expressing lentiviral particles (GeneCopoeia) were generated using highly purified plasmids and EndoFectin-Lenti (GeneCopoeia) and TiterBoost (GeneCopoeia) reagents following a standardized protocol. OMSCs were transfected using polybrene (GeneCopoeia), and 12 hours posttransfection, the medium was replaced with complete media; 36 hours posttransfection, the medium was replaced with osteogenic media. For siRNA transfection, Gli1-siRNA and Gli2-siRNA were synthesized according to a standardized protocol (Hippobio, Co., Ltd.**)**. OMSCs were transfected with Lipofectamine 3000 (Invitrogen) for 48 hours prior to experimentation, followed by replacement of the medium with osteogenic media. Sequences are listed in Supplemental Table 3.

### Collection of human alveolar bone samples.

The collection of human samples was permitted by the Ethical Committees of the West China Hospital of Stomatology, Sichuan University (WCHSIRB-D-2021-292). Written informed consent was obtained from every patient. Bony samples from AP lesions were collected from discards during endodontic surgery. Normal alveolar bone tissue was harvested from patients receiving mandibular osteotomy surgery. There was no significant difference in age or sex between patients in 2 groups. The samples were rinsed with sterile PBS, followed by quick-freezing of some samples in liquid nitrogen for RNA extraction. The remaining samples were fixed in 4% PFA and decalcified for immunostaining.

### Statistics.

Prism 8 (GraphPad Software Inc.) was used for statistical analysis. Unpaired 2-tailed Student’s *t* test was used in 2-group comparisons. One-way ANOVA or 2-way ANOVA was performed for multiple comparisons. Data represent mean ± SEM. A *P* value less than 0.05 was considered significant.

### Study approval.

Animal experiments were conducted in accordance with the protocol approved by Institutional Animal Care and Use Committee at the State Key Laboratory of Oral Diseases, Sichuan University (WCHSIRB-D-2021-339). The collection of human samples was permitted by the Ethical Committees of the West China Hospital of Stomatology, Sichuan University (WCHSIRB-D-2021-292). Written informed consent was received prior to participation.

### Data availability.

Sequence data have been deposited in the National Center for Biotechnology Information Gene Expression Omnibus under accession numbers GSE212975 and GSE221990. Values for all data points in graphs are reported in the Supporting Data Values file.

## Author contributions

YF, CJR, and CZ designed all experiments and wrote the manuscript. Experiments were performed by YF, CZ, PL, JW, YW, and ZL. PL, SZ, JJ, and QY performed and analyzed micro-CT and histomorphometry data. YF, CJR, PL, and TO analyzed RNA-Seq data. YF, CJR, CZ, TO, and QY provided reagents and planned experiments. The order of co–first authors YF and PL was determined by their contribution to the article. YF designed experiments, conducted the experiments, wrote the manuscript, and analyzed the data. PL conducted experiments and analyzed the data. All authors edited and approved the manuscript.

## Supplementary Material

Supplemental data

Supporting data values

## Version 1 12/17/2024

In-Press Preview

## Version 2 02/10/2025

Electronic publication
