# Supplementary material for: Negative feedback between PTH1R and IGF1 through the Hedgehog pathway in mediating craniofacial bone remodeling
Source: JCI Insight. 2024 Dec 17;10(3):e183684. doi: 10.1172/jci.insight.183684 (PMC11948590; doi:10.1172/jci.insight.183684)
Supplement: Supplemental data [file jciinsight-10-183684-s062.pdf]

Supplemental data

Supplemental Figures

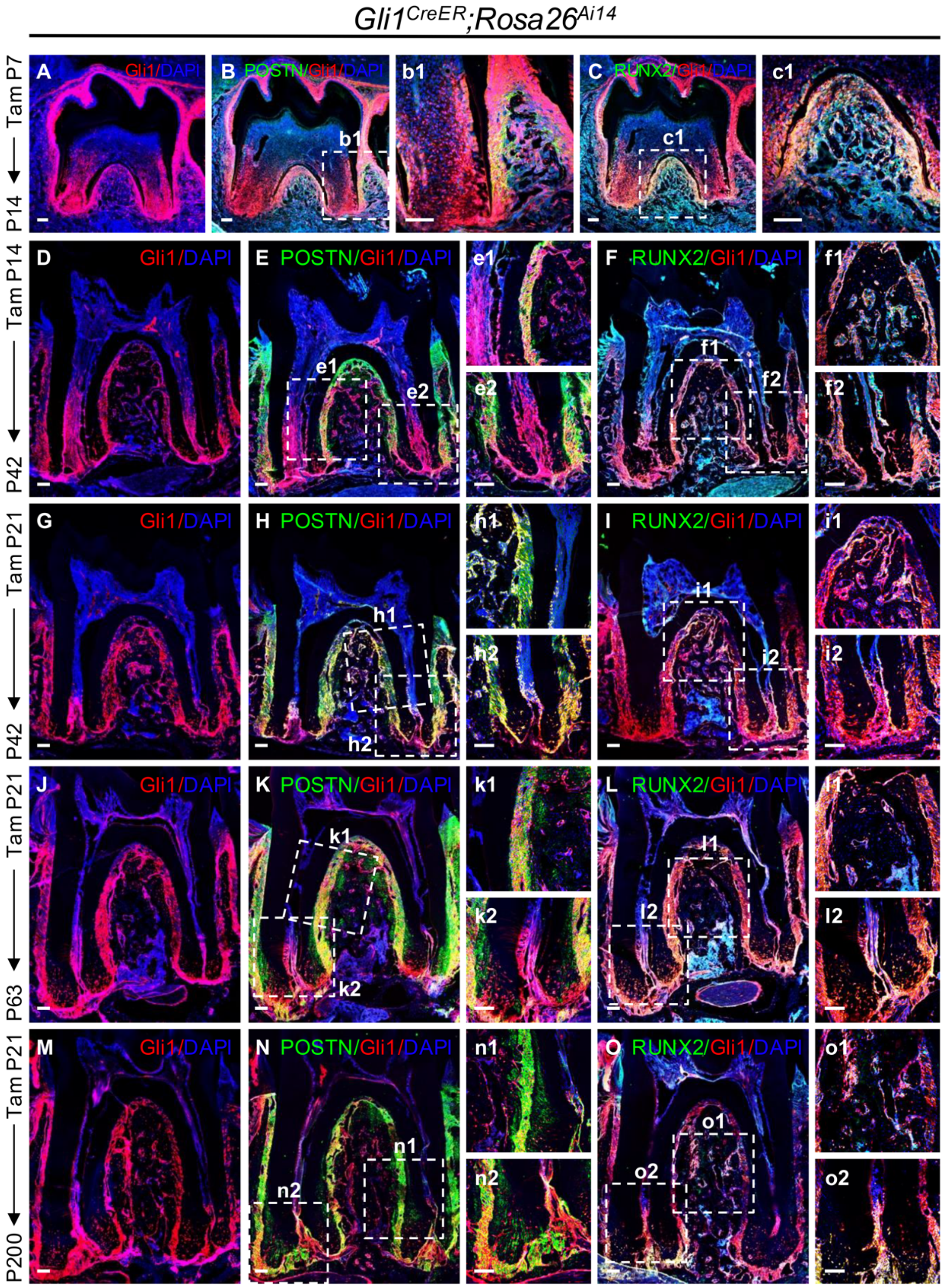

Supplemental Figure 1. Gli1^{+} cells distribution in apical pulp, PDL, and oral bone.

(**A-C**) Lineage tracing of Gli1$^+$ lineage cells on postnatal day 14 (P14) after Tamoxifen induction at P7. Immunofluorescent staining of Periostin (POSTN) (**B**), and RUNX2 (**C**) was performed. Boxed areas are shown at higher magnification in (**b1**), (**c1**). n=3.

(**D-F**) Lineage tracing of Gli1$^+$ lineage cells on P42 after Tamoxifen induction at P14. Immunofluorescent staining of POSTN (**E**), and RUNX2 (**F**) was performed. Boxed areas are shown at higher magnification in (**e1**), (**e2**), (**f1**), (**f2**). n=3. The osteocyte number was calculated in the furcation area of the first mandibular molars in at least six confocal images of each sample.

(**G-I**) Lineage tracing of Gli1$^+$ lineage cells on P42 after Tamoxifen induction at P21. Immunofluorescent staining of POSTN (**H**), and RUNX2 (**I**) was performed. Boxed areas are shown at higher magnification in (**h1**), (**h2**), (**i1**), (**i2**). n=3.

(**J-L**) Lineage tracing of Gli1$^+$ lineage cells on P63 after Tamoxifen induction at P21. Immunofluorescent staining of POSTN (**K**), and RUNX2 (**L**) was performed. Boxed areas are shown at higher magnification in (**k1**), (**k2**), (**l1**), (**l2**). n=3.

(**M-O**) Long-term lineage tracing of Gli1$^+$ lineage cells P200 after Tamoxifen induction at P21. Immunofluorescent staining of POSTN (**N**), and RUNX2 (**O**) was performed. Boxed areas are shown at higher magnification in (**n1**), (**n2**), (**o1**), (**o2**). n=3.

Scale bar=100 μm.

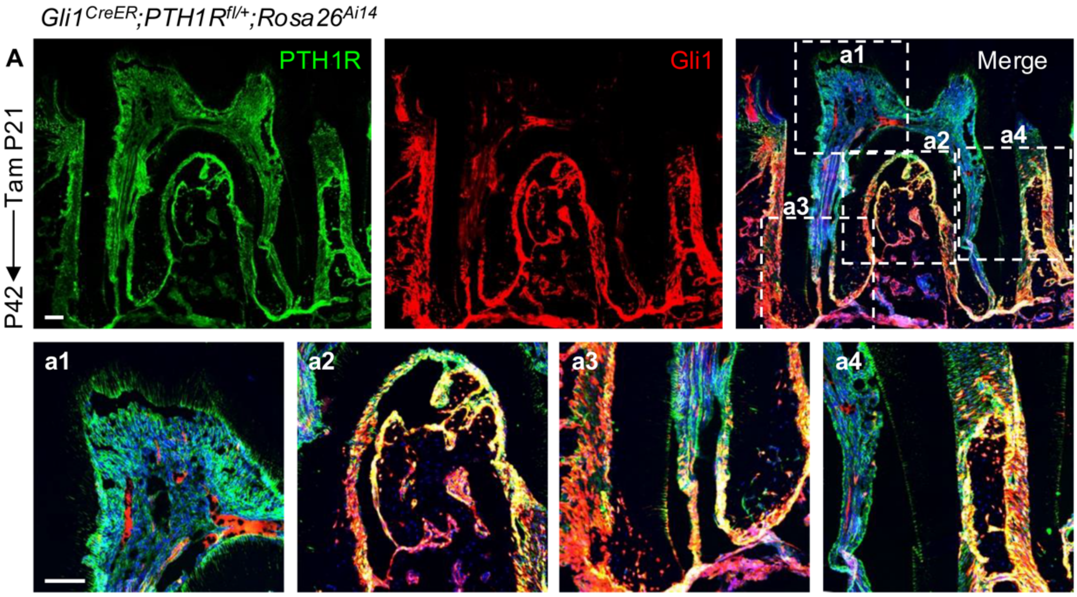

Supplemental Figure 2. Distribution of PTH1R and Gli1$^+$ lineage cells.

(**A**) Immunofluorescent staining of PTH1R in *Gli1^{CreER};PTH1R$^{fl/+}$;Rosa26^{Ai14}* mice showed co-localization of PTH1R and Gli1$^+$ lineage cells. Boxed areas are shown at higher magnification. n=3. Scale bar=100 μm.

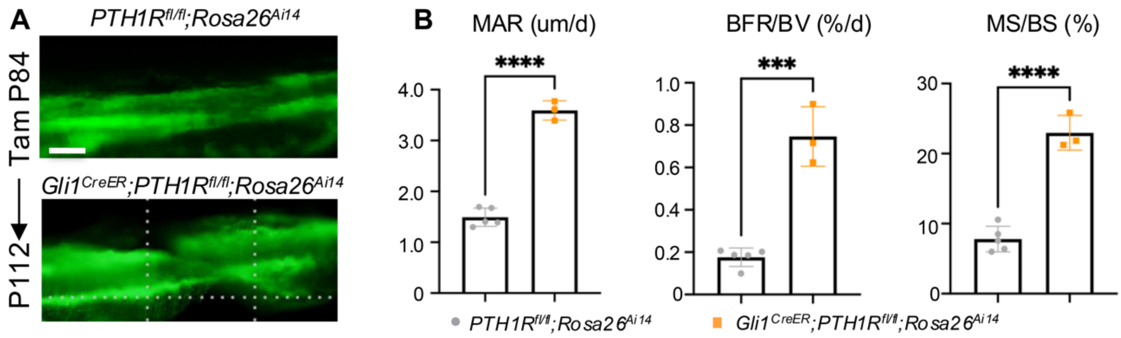

Supplemental Figure 3. Bone histomorphometry analysis at P112.

(**A**) Double calcein labeling in the alveolar bone region of control and PTH1R-cKO mice at P112.

(**B**) Histomorphometry analysis of dynamic bone formation parameters at P112. n=5 in control and n=3 in PTH1R-cKO mice. Male mice were used. Data are mean ± SEM. ***P<0.001, ****P< 0.0001.

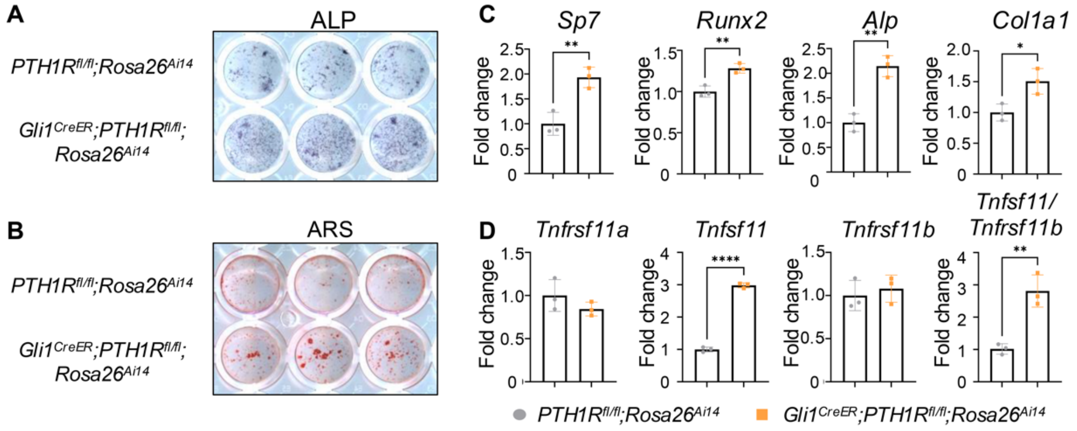

Supplemental Figure 4. PTH1R-deficient OMSCs presented increased osteogenic differentiation potential.

(**A-B**) Alkaline phosphatase (ALP) staining at 7-day of osteogenic induction and alizarin red staining (ARS) at 14-day of osteogenic induction reveal a significantly increased osteogenesis of OMSCs extracted from *Gli1^{CreER};PTH1R$^{fl/fl}$;Rosa26^{Ai14}* mice. n=3.

(**C, D**) RT-qPCR analysis showed upregulated osteogenic-related gene expression (*Sp7, Runx2, Alp, Col1a1, Tnfsf11*) of PTH1R-cKO OMSCs after 14 d of osteogenic induction. n=3.

Significance is determined using unpaired two-sided student's t-tests. Data are mean ± SEM. **P*<0.05, ***P*<0.01, *****P*<0.0001.

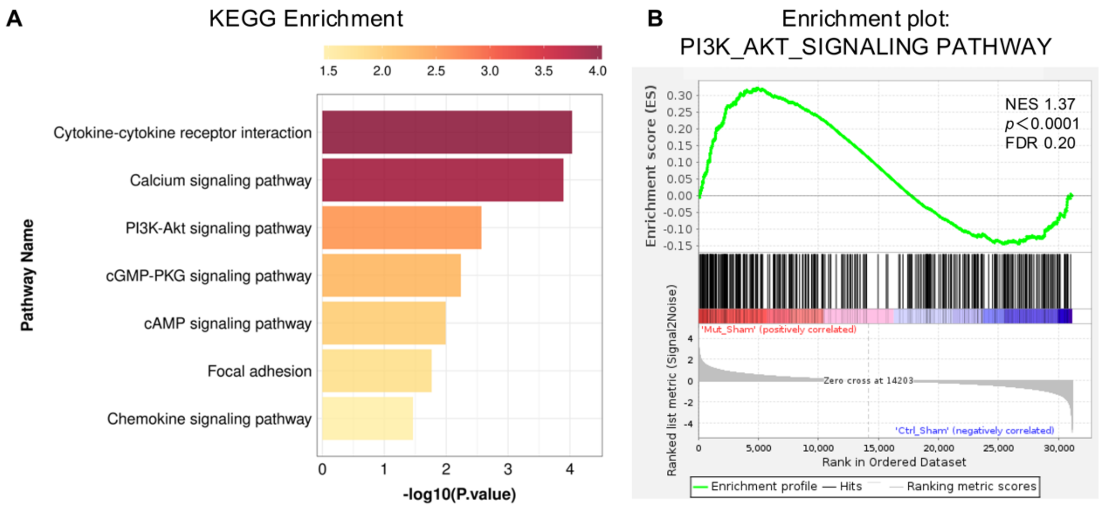

Supplemental Figure 5. KEGG enrichment analysis of control and PTH1R-cKO mice.

(**A**) KEGG enrichment result bar graph of PTH1R-cKO vs. control mandibular bones.

(**B**) GSEA plots showed the upregulation of PI3K-Akt signaling pathway in PTH1R-cKO mice.

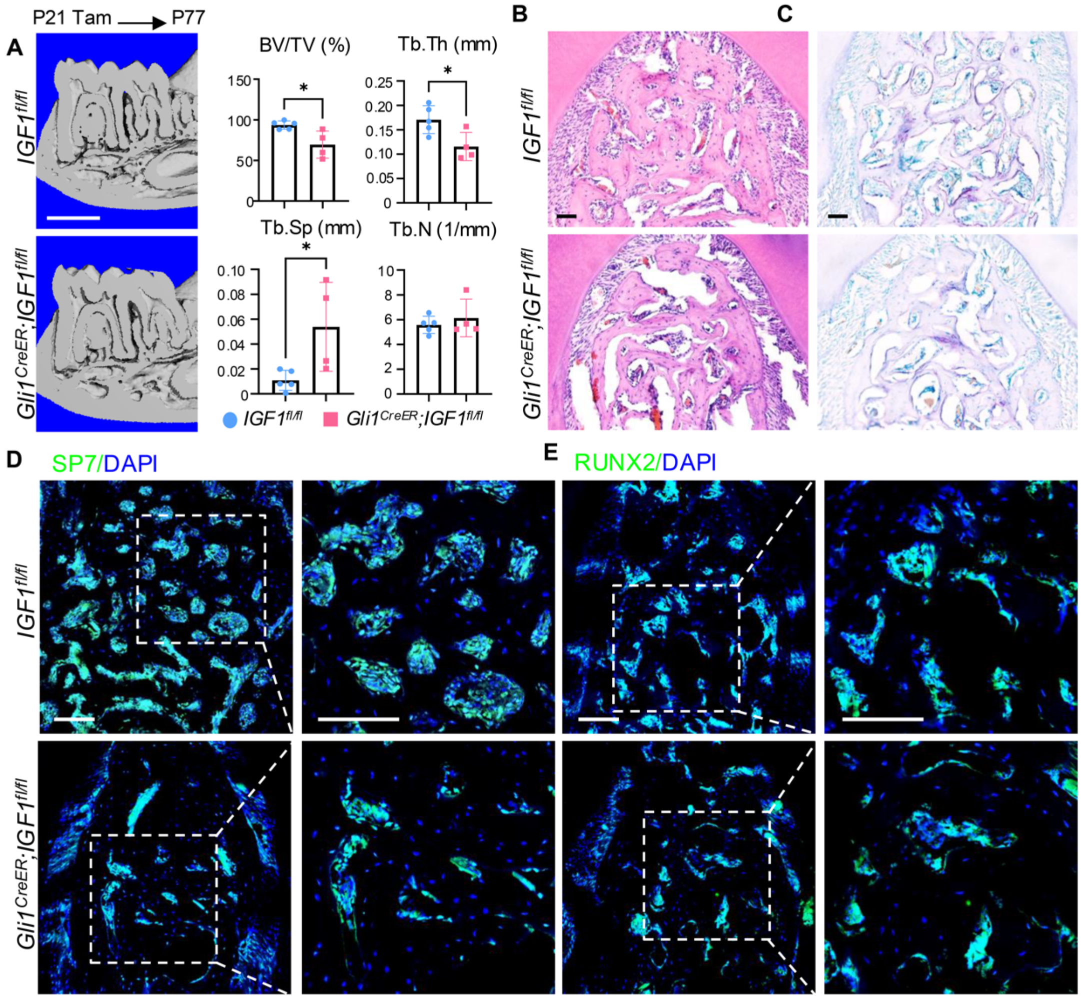

Supplemental Figure 6. *Gli1^{CreER};IGF1$^{fl/fl}$* mice exhibit low bone turnover.

(**A**) Three-dimensional micro-CT reconstruction and quantitative analysis of alveolar bone parameters including BV/TV (%), Tb. Th (mm), Tb. Sp (mm) and Tb. N (1/mm) of male control and IGF1-cKO mice. n=5 for *IGF1$^{fl/fl}$* and n=4 for *Gli1^{CreER};IGF1$^{fl/fl}$* mice.

(**B**) HE staining of mandibular first molar furcation area showed reduced bone volume of IGF1-cKO mice. n=3.

(**C**) TRAP staining displayed reduced TRAP^{+} osteoclasts in IGF1-cKO mice. n=3.

(**D, E**) Immunofluorescent staining of SP7 (**D**) and RUNX2 (**E**) revealed decreased Sp7^{+} and Runx2^{+} osteoblast numbers in IGF1-cKO mice alveolar bone. Boxed areas are shown at higher magnification. n=3.

Significance is determined using unpaired two-sided student's t-tests. Data are mean ± SEM. *P<0.05. Scale bar=1 mm in A, 100 μm (B-E).

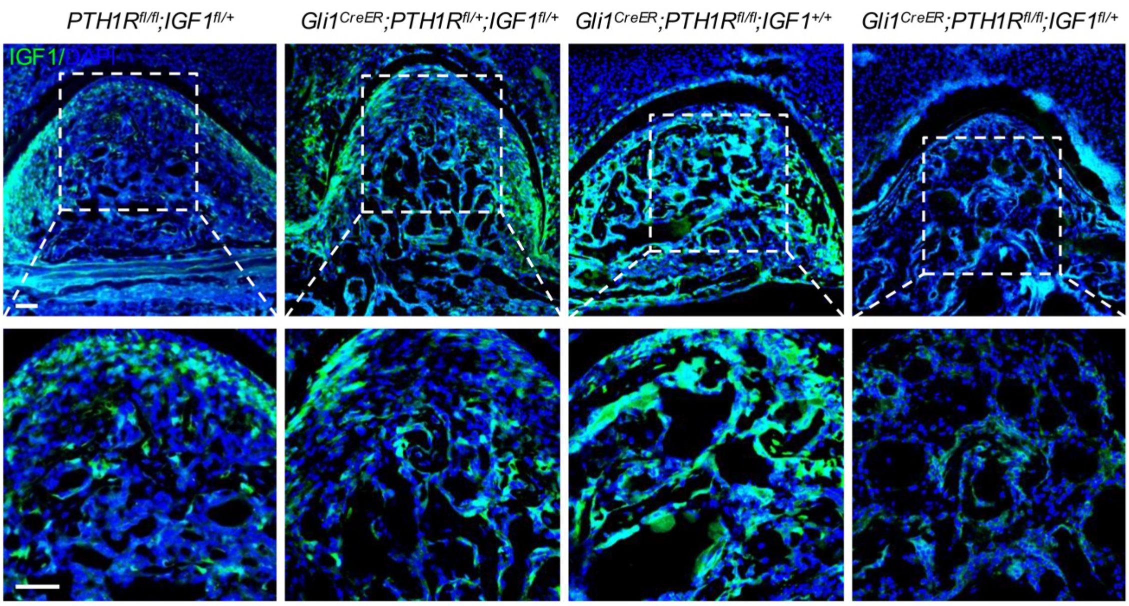

Supplemental Figure 7. IGF1 expression in alveolar bone of PTH1R and IGF1 deficient mouse models.

Immunofluorescent staining revealed IGF1 expression was increased in alveolar bone of PTH1R-cKO mice, which was reversed in *Gli1*^{CreER}*;PTH1R*$^{fl/fl}$*;IGF1*$^{fl/+}$ mice. Boxed areas are shown at higher magnification. n=3.

Scale bar=100 μm in lower magnification and 200 μm in higher magnification.

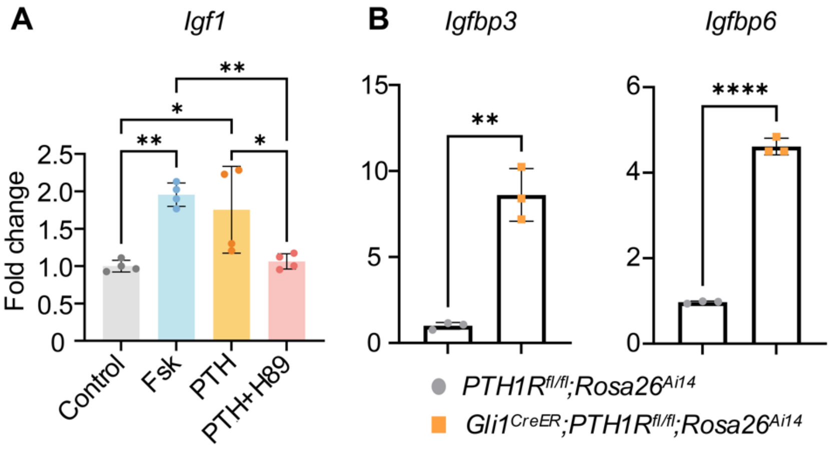

Supplemental Figure 8. Gene expression levels of *Igf1* and *Igfbps*.

(**A**) Pre-osteoblast cell line MC3T3-E1 were treated with vehicle, 100 nM PTH (1-34) (Bachem), 20 μM Forskolin (Fsk) (Selleck), 100 nM PTH + 10 μM H89 (Selleck) respectively, for 6 hours prior to RNA extraction. RT-qPCR showed *Igf1* expression upon Fsk, PTH (1-34), or PTH (1-34)+H89 treatment. n=4.

(**B**) RT-qPCR analysis of *Igfbp3* and *Igfbp6* gene expression in OMSCs extracted from control or PTH1R-cKO mice. n=3.

Significance is determined using one-way ANOVA with Tukey's correction in **A** and unpaired two-sided student's t-tests in **B**. Data are mean ± SEM. *P<0.05, **P<0.01, ****P<0.0001.

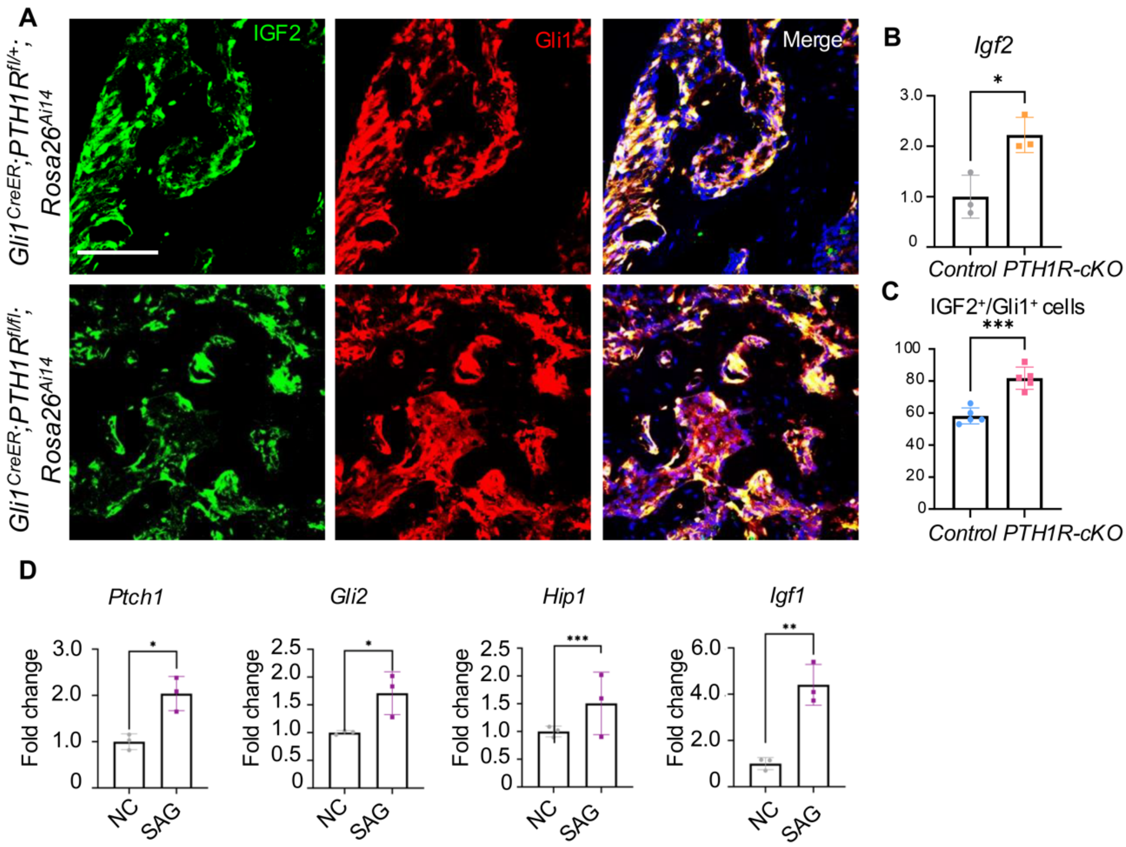

Supplemental Figure 9. IGF2 expression in control and PTH1R-cKO mice and Hedgehog signaling related gene expressions following treatment with Smoothened Agonist (SAG).

(**A, C**) Immunofluorescent staining and quantification showed increased $IGF2^{+}/Gli1^{+}$ cell number in root furcation area of *Gli1^{CreER};PTH1R$^{fl/fl}$;Rosa26^{Ai14}* female mice at P42. n=5.

(**B**) RT-qPCR results showed upregulated *Igf2* in PTH1R-cKO OMSCs. n=3.

(**D**) Gene expression levels of Hedgehog transcriptional targets (*Ptch1*, *Gli2*, and *Hip1*) and *Igf1* in OMSCs at 48 hours post SAG treatment. Significance is determined using unpaired two-sided student's t-tests. Data are mean ± SEM. *P<0.05, **P<0.01, ***P<0.001.

Supplemental Tables.

Supplemental Table 1. Mouse genotyping primers.

**Gene**	**Forward primer**	**Reverse primer**	**Expected**
Rosa26^{Ai14}	GGCATTAAAGCAGCGTATCC	CTGTTCCTGTACGGCATGG	196 bp
WT	AAGGGAGCTGCAGTGGAGTA	CCGAAAATCTGTGGGAAGTC	297 bp
Gli1^{CreER}	GCGGTCTGGCAGTAAAAACTATC	GTGAAACAGCATTGCTGTCACTT	100 bp
WT	CTAGGCCACAGAATTGAAAGATCT	GTAGGTGGAAATTCTAGCATCATCC	136 bp
IGF1 mutant	AAACCACACTGCTCGACATTG	CACTAAGGAGTCTGTATTTGGACC	275 bp
WT	GGCAAATGGAAATCCTATGTCT	CACTAAGGAGTCTGTATTTGGACC	398 bp
PTH1R	ATGAGGTCTGAGGTACATGGCTCTGA	CCTGCTGACCTCTCTGAAAGAATGT	280 bp (mutant)<br>210 bp (WT)

Supplemental Table 2. RT-qPCR primer sequences.

Gene	Forward primer	Reverse primer
Mouse *Actin*	AAGGCCAACCGTGAAAAGAT	GTGGTACGACCAGAGGCATAC
Mouse *Pth1r*	CCAGCATCTACGTCAGGGAAG	CAAACGATGTTGTCCCACTCT
Mouse *Sp7*	CTCCTTGGTGGGACATGC	GTAGGCAGCTGGGGGTTC
Mouse *Runx2*	TCCACAAGGACAGAGTCAGATTACAG	CAGAAGTCAGAGGTGGCAGTGTCATC
Mouse *Alp*	CACGGCCATCCTATATGGTAA	GGGCCTGGTAGTTGTTGTGA
Mouse *Opn*	ATCTCACCATTCGGATGAGTCT	TGTAGGGACGATTGGAGTGAAA
Mouse *Ctsk*	CAGCTTCCCCAAGATGTGAT	AAAAATGCCCTGTTGTGTCC
Mouse *Mmp9*	CTGGACAGCCAGACACTAAAG	CTCGCGGCAAGTCTTCAGAG
Mouse *Nfatc1*	TGCCTTTTGCGAGCAGTATCT	CAGGCAAGGATGGGCTCATAT
Mouse *Atp6v0d2*	CAGAGCTGTACTTCAATGTGGAC	AGGTCTCACACTGCACTAGGT
Mouse *Rank*	CCAGGAGAGGCATTATGAGCA	ACTGTCGGAGGTAGGAGTGC
Mouse *Rankl*	GCAGAAGGAACTGCAACACA	GATGGTGAGGTGTGCAAATG
Mouse *Opg*	CAGCATCGCTCTGTTCCTGTA	CTGCGTTTTCATGGAGTCTCA
Mouse *Col1a1*	GCGCTAAAGGTGCCAATG	AGCACCAGGTTCACCACTG
Mouse *Igf1*	CACATCATGTCGTCTTCACACC	GGAAGCAACACTCATCCACAATG
Mouse *Igf2*	GTGCTGCATCGCTGCTTAC	CGGTCCGAACAGACAAACTG
Mouse *Ptch1*	AAAGAACTGCGGCAAGTTTTTG	CTTCTCCTATCTTCTGACGGGT
Mouse *Smo*	GTGCTGTCTACATGCCCAAGT	GCAACGCAGAAAGTCAGGC
Mouse *Gli2*	CAACGCCTACTCTCCCAGAC	GAGCCTTGATGTACTGTACCAC
Mouse *Hip1*	GGTCAGCGTCAATAAGGCCAT	GGTCTGCGCTCCTTTCTCA

Mouse *Igfbp3*	CACACCGAGTGACCGATTCC	GTGTCTGTGCTTTGAGACTCAT
Mouse *Igfbp6*	TGCTAATGCTGTTGTTCGCTG	CACGGTTGTCCCTCTCTCCT
Human *GAPDH*	ACTGAGGACCAGGTTGTC	TGCTGTAGCCGTATTCATTG
Human *PTH1R*	CAGGCGCAATGTGACAAGC	TTTCCCGGTGCCTTCTCTTTC
Human *IGF1*	CACATCATGTCGTCTTCACACC	GGAAGCAACACTCATCCACAATG

Supplemental Table 3. Sequences for RNAi experiments.

Igf1 shRNA target sequence	TATTATTGTTATTTGGTAGGTG
NC shRNA hairpin loop sequence	TAGTGAAGCCACAGATGTA
Gli1 siRNA sequence	CCCAACAUGGAGUGGGUAACAUGA<br>UCAUGUUACCCACUGCCAUGUUGGG
Gli2 siRNA sequence	CCACAACCACAACGUUGCUCAGACA<br>UGUCUGAGCAAGCUUGUGGUUGUGG
